# The power new XLindley distribution: Statistical inference, fuzzy reliability, and applications

**DOI:** 10.1016/j.heliyon.2024.e36594

**Published:** 2024-08-22

**Authors:** Ahmed M. Gemeay, Abdelali Ezzebsa, Halim Zeghdoudi, Caner Tanış, Yusra A. Tashkandy, M.E. Bakr, Anoop Kumar

**Affiliations:** a Department of Mathematics, Faculty of Science, Tanta University, Tanta 31527, Egypt; b LaPS laboratory, Badji Mokhtar -Annaba University, Annaba, Algeria and 8 May 1945 University, Guelma, Algeria; c LaPS laboratory, Badji Mokhtar - Annaba University, Annaba, Algeria; d Department of Statistics, Çankırı Karatekin University, Çankırı, Turkey; e Department of Statistics and Operations Research, College of Science, King Saud University, P.O. Box 2455, Riyadh 11451, Saudi Arabia; f Department of Statistics, Faculty of Basic Science, Central University of Haryana, Mahendergarh, 123031, India

**Keywords:** 60E05, 62E10, 62N05, Lindley distribution, Moments, Estimation, Fuzzy

## Abstract

This paper introduces the power new XLindley (PNXL) distribution, a novel two-parameter distribution derived using the power transformation method applied to the XLindley distribution. We thoroughly explore the structural properties of the PNXL distribution, including the *r*th moment about the origin, moment generating function, survival rate function, distribution function, hazard rate function, skewness, kurtosis, and coefficient of variation. Additionally, we derive the quantile function, fuzzy reliability, reliability measures, stochastic ordering, and actuarial measures for this new distribution. To estimate the parameters of the PNXL distribution, we propose several estimators and evaluate their performance through extensive simulation studies. To demonstrate the applicability and superiority of the PNXL distribution over existing distributions, we fit it to two real datasets and compare its performance with potential competing models. The results highlight the PNXL distribution's effectiveness and potential as a robust tool for modeling and analyzing real-world data.

## 1 Introduction

The usefulness of lifetime distributions in many fields, such as health, economics, agriculture, engineering, meteorology, etc., has been demonstrated in many studies in the literature. One-parameter distributions are very popular in the literature. Adding parameters to the current distribution allows the distribution to be flexible regarding data modeling. Therefore, many authors have tried to improve the flexibility of existing distributions and proposed new distributions by new parameterization. Some of the studies in the literature, including newly generated distributions with new parameterizations, are listed as follows: The gamma distribution, the two-parameter Lindley distribution I (see Shanker et al. [17]), the gamma Lindley distribution (see Zeghdoudi and Nedjar [25], [13]), the quasi Lindley distribution (see Benatmane et al. [4]), the new quasi Lindley (see Shanker and Ghebretsadik [16]), and the pseudo-Lindley distribution (see Zeghdoudi and Nedjar [26]). Consequently, the two-parameter distributions model several phenomena. Other studies have constructed more adaptable Lindley generalizations by combining Lindley with other well-known distributions. Researchers in [18] and [19] examined a two-parameter extension of the Lindley distribution.

Recently, Khodja et al. [12] introduced a new statistical distribution named the new XLindley distribution based on a special mixture of exponential and Lindley distributions. Its probability density function (PDF) is defined as follows

$$f_{NXL}(y;\gamma )=\frac{\gamma }{2}\left(1+\gamma y\right)\exp\left(-\gamma y\right),\;\;y,\gamma >0,$$

and its cumulative distribution function (CDF) is defined as follows

$$F_{NXL}(y;\gamma )=1-\left(\frac{1}{2}\gamma y+1\right)e^{-\gamma y},\;\;y,\gamma >0.$$

On the other hand, some of the suggested two parameters distributions by using the power transformation are listed as follows: power Shanker [18], power Aradhana [19], power Sujatha [20], Power Prakaamy [21], and power Zeghdoudi [2]. All the power-transformed distributions were shown to be more adaptable than their respective baseline distributions and more effective for analyzing complicated data structures in various spheres of life, according to the literature reviewed in this paper.

The motivation of this work is to obtain a new distribution that is more flexible than the NXL distribution, called the PNXL distribution, using the power transformation method.

We investigate the shapes of the density and hazard rate functions, the moments and some associated measures, the quantile function, stochastic ordering, the limiting distributions of order statistics, fuzzy reliability, and actuarial measures. The estimation methods for our model are discussed using several different estimation methods. Many data sets are presented to demonstrate the proposed distribution's flexibility and compare it to the fit that some other well-known distributions achieve. This work has been affected by the following factors:

• The application of the PNXL distribution may be limited to the tail of a distribution, but it is simple to use.
• The statistical properties are easily determined in an explicit form.
• The novel distribution has advantages, such as having two parameters that allow us to model survival analysis, actuarial science, etc.
• The PNXL distribution can be used quite effectively in analyzing numerous real-life data sets and adequately fits these data sets.
• The PNXL distribution's density function can be increasing, decreasing, or unimodal.
• The distribution's hazard function can be decreasing, increasing, or decreasing—increasing—decreasing.

The paper is organized as follows. In Section 2, we present the formulation of the PNXL distribution. In Section 3, we introduce different statistical properties of the PNXL distribution. Section 4 provides fuzzy reliability of the PNXL distribution. In Section 5, we describe some actuarial measures for the proposed distribution. In Section 6, we discuss sixteen estimation methods to estimate the parameters of the PNXL distribution. Section 7 considers a simulation study to compare these estimation methods. Section 8 analyses the fit of PNXL distributions and their competitors to real-world data sets.

## 2 Formulation of PNXL distribution

In this section, we provide the PDF, survival function (SF), and hazard rate function (HRF) of the PNXL distribution. The Lindley, XLindley, and new XLindley distributions may not be appropriate for many theoretical problems. To have a flexible model, we derived the power new XLindley (PNXL) distribution which is based on the power transformation $T=Y^{\frac{1}{\gamma }}$. The *T* PDF can be obtained as follows:

(1) $$f_{PNXL}(t;\beta ,\gamma )=\frac{1}{2}\beta \gamma t^{\gamma -1}\left(\beta t^{\gamma }+1\right)e^{-\beta t^{\gamma }}=\frac{1}{2}f_{1}(t)+\frac{1}{2}f_{2}(t),\;\;\;x,\beta ,\gamma >0,$$

where $f_{1}(t)=\beta \gamma t^{\gamma -1}e^{-\beta t^{\gamma }}$ follows Weibull distribution with shape parameter *γ* and scale parameter *β*, and $f_{2}(t)=\gamma \beta^{2}t^{2\gamma -1}e^{-\beta t^{\gamma }}$ follows a generalized gamma distribution with shape parameters 2 and *γ* and scale *β*. So, we can say that The PDF PNXL distribution (1) is a two-component mixture of Weibull distribution and a generalized gamma distribution.

The SF and HRF of PXL distribution are, respectively, defined as follows

(2) $$S_{PNXL}(t;\beta ,\gamma )=\left(\frac{1}{2}\beta t^{\gamma }+1\right)e^{-\beta t^{\gamma }},\;\;x,\beta ,\gamma >0,h_{PNXL}(t;\beta ,\gamma )=\frac{f_{PNXL}(t;\beta ,\gamma )}{S_{PNXL}(t;\beta ,\gamma )}=\frac{\beta \gamma t^{\gamma -1}\left(\beta t^{\gamma }+1\right)}{\left(\beta t^{\gamma }+2\right)}.$$

## 3 Statistical properties

In this section, many statistical properties are presented, such as the behavior of PDF, HRF, and quantile function, as well as moments, incomplete moments, stochastic ordering, and limitations of order statistics.

### 3.1 Asymptotic behavior

This subsection discusses the behavior and possible shape characteristics of the PDF $f_{PNXL}(t;\beta ,\gamma )$ and HRF $h_{PNXL}(t;\beta ,\gamma )$ in Equations (1) and (2), respectively, of the PXL distribution. The PDF behavior is described as follows

$$\lim_{t\to 0}f_{PNXL}(t;\beta ,\gamma )=\left\{ \begin{array}{cc} \infty \; & \text{if }\gamma <1\\ \frac{\beta }{2}\; & \text{if }\gamma =1\\ 0\; & \text{if }\gamma >1 \end{array},\right.\lim_{t\to \infty }f_{PNXL}(t;\beta ,\gamma )=0.$$

The behavior of $h_{PNXL}(t;\beta ,\gamma )$ at t=0 and t=∞, respectively, are given by

$$\lim_{t\to 0}h_{PNXL}(t;\beta ,\gamma )=\left\{ \begin{array}{cc} \text{Indeterminate}\; & \text{if }\gamma <1\\ \frac{\beta \gamma }{2}\; & \text{if }\gamma =1\\ 0\; & \text{if }\gamma >1 \end{array},\right.\lim_{t\to \infty }h_{PNXL}(t;\beta ,\gamma )=\left\{ \begin{array}{cc} 0\; & \text{if }\gamma <1\\ \beta \; & \text{if }\gamma =1\\ \infty \; & \text{if }\gamma >1 \end{array}\right..$$

The following proposition states that there are three shapes for the PDF of the power new XLindley distribution, depending on the range of the parameters *γ* and *β*.

Proposition 1 *The PDF f* ${}_{PNXL}(t;\beta ,\gamma )$ *in* (1) *of the PNXL distribution is*

1. *Decreasing if* 0<γ≤1 *,* β>0 *.*
2. *Unimodal if* γ>1,β>0 *and the mode is* ${\overset{ˆ}{t}}_{0}={\left[\frac{1}{2\beta \gamma }\left(\gamma +\sqrt{-6\gamma +5\gamma^{2}+1}-1\right)\right]}^{\frac{1}{\gamma }}$ *.*

Proof The first derivative of $f_{PNXL}(t;\beta ,\gamma )$ is

$$\frac{df_{PNXL}(t;\beta ,\gamma )}{dt}=\frac{1}{2}t^{\gamma -2}\beta \gamma e^{-\beta t^{\gamma }}L\left(t^{\gamma }\right),$$

where $L\left(t^{\gamma }\right)=at^{2\gamma }+bt^{\gamma }+c$, with $a=-\beta^{2}\gamma$, b=β(γ−1), and c=γ−1. We can see that $\frac{df_{PNXL}(t;\beta ,\gamma )}{dt}$ and $L\left(t^{\gamma }\right)$ have the same sign. Using the characteristics of the quadratic function $L\left(t^{\gamma }\right)$ under the previous conditions in (1), and (2), the function $L\left(t^{\gamma }\right)$ is negative for γ≤1, unimodal with maximum value at the point $t_{0}^{\gamma }=\frac{1}{2\beta \gamma }\left(\gamma +\sqrt{-6\gamma +5\gamma^{2}+1}-1\right)$ for γ>1 and the mode is

$${\overset{ˆ}{t}}_{0}={\left[\frac{1}{2\beta \gamma }\left(\gamma +\sqrt{-6\gamma +5\gamma^{2}+1}-1\right)\right]}^{\frac{1}{\gamma }},$$

and $\frac{d^{2}f_{PNXL}({\overset{ˆ}{t}}_{0};\beta ,\gamma )}{dt^{2}}<0$. This completes the proof of Proposition 1. □

The following proposition showed the different shapes for the HRF of PNXL distribution, depending on the values of the parameters *γ* and *β*.

Proposition 2 *The HRF* $h_{PNXL}(t;\beta ,\gamma )$ *in* (2) *of the PNXL distribution is*

1. *Decreasing if*$0<\gamma \leq \frac{3}{4},\beta >0$*, and γ*= $\frac{\sqrt{2}}{4}+\frac{1}{2}$*.*
2. *Increasing if* γ≥1,β>0 *.*
3. *Decreasing–increasing–decreasing if* $\frac{3}{4}<\gamma <\frac{\sqrt{2}}{4}+\frac{1}{2},\beta >0$ *and* $\frac{\sqrt{2}}{4}+\frac{1}{2}<\gamma <1,\beta >0$ *.*

Proof The first derivative of $h_{PNXL}(t;\beta ,\gamma )$ is

$$\frac{dh_{PNXL}(t;\beta ,\gamma )}{dt}=\frac{\beta \gamma t^{\gamma -2}}{{\left(t^{\gamma }\beta +2\right)}^{2}}M(t^{\gamma }),$$

where $M(t^{\gamma })=\beta^{2}\left(\gamma -1\right)t^{2\gamma }+\beta \left(4\gamma -3\right)t^{\gamma }+2\left(\gamma -1\right)$

We can see that $\frac{dh_{PNXL}(t;\beta ,\gamma )}{dt}$ and $M\left(t^{\gamma }\right)$ have the same sign. Using the characteristics of the quadratic function $M\left(t^{\gamma }\right)$ under the previous conditions in (1), (2), and (3), the function $M\left(t^{\gamma }\right)$ is negative for $0<\gamma \leq \frac{3}{4}$, and *γ*= $\frac{\sqrt{2}}{4}+\frac{1}{2}$, positive for γ≥1, and negative-positive-negative for $\frac{3}{4}<\gamma <\frac{\sqrt{2}}{4}+\frac{1}{2}$, and $\frac{\sqrt{2}}{4}+\frac{1}{2}<\gamma <1$. The rest of the proof of this proposition follows similarly to that of Proposition 1. □

### 3.2 The quantile function of PNXL distribution

In this subsection, we present the quantile function of PNXL distribution. According to Khodja et al. [12], the quantile function of the new XLindley distribution is given by

$$Q_{Y}(u)=VaR=y_{u}=-\frac{2}{\beta }-\frac{1}{\beta }W_{-1}\left[\frac{2(u-1)}{e^{2}}\right],\;u\in \left[0,1\right],$$

where $W_{-1}($·) is the negative branch Lambert function.

Then, The PNXL distribution quantile function is given by

$$F_{T}^{-1}(u)={\left[-\frac{2}{\beta }-\frac{1}{\beta }W_{-1}\left[\frac{2(u-1)}{e^{2}}\right]\right]}^{\frac{1}{\gamma }}.$$

### 3.3 Moments and associated measures

This subsection provides some statistical measures of PNXL distribution such as moments, mean, variance, skewness, kurtosis, coefficient of variation (C.V), moment generation function and incomplete moments.

The $r^{th}$ moment of the PNXL distribution can be obtained as:

$$\mu_{r}^{\prime }=E(T^{r})=\int_{0}^{\infty }t^{\left(r\right)}f_{PNXL}(t)dt=\frac{1}{2}\frac{\Gamma \left(\frac{r}{\alpha }+1\right)}{\beta^{\frac{r}{\alpha }}}+\frac{1}{2}\frac{\Gamma \left(\frac{r}{\alpha }+2\right)}{\beta^{\frac{r}{\alpha }}}=\frac{r\left(r+2\gamma \right)\Gamma \left(\frac{r}{\gamma }\right)}{2\gamma^{2}\beta^{\frac{r}{\alpha }}}.$$

The mean, variance, C.V, skewness, and kurtosis of the PNXL distribution are respectively given by

$$E(T)=\frac{\left(2\gamma +1\right)\Gamma \left(\frac{1}{\gamma }\right)}{2\beta^{\frac{1}{\alpha }}\gamma^{2}},Var(T)=\frac{\left(2\gamma +2\right)\Gamma \left(\frac{2}{\gamma }\right)}{\beta^{\frac{2}{\alpha }}\gamma^{2}}-\frac{{\left(2\gamma +1\right)}^{2}{\left(\Gamma \left(\frac{1}{\gamma }\right)\right)}^{2}}{4\beta^{\frac{2}{\alpha }}\gamma^{4}},$$

$$Skewness=\sqrt{\beta_{1}}=\frac{E(T^{3})}{{\left(Var(T)\right)}^{\frac{3}{2}}}=\frac{\frac{3}{2\beta^{\frac{3}{\alpha }}\gamma^{2}}\Gamma \left(\frac{3}{\gamma }\right)\left(2\gamma +3\right)}{{\left(\frac{\left(2\gamma +2\right)\Gamma \left(\frac{2}{\gamma }\right)}{\beta^{\frac{2}{\alpha }}\gamma^{2}}-\frac{{\left(2\gamma +1\right)}^{2}{\left(\Gamma \left(\frac{1}{\gamma }\right)\right)}^{2}}{4\beta^{\frac{2}{\alpha }}\gamma^{4}}\right)}^{\frac{3}{2}}},$$

$$Kurtosis=\beta_{2}=\frac{E(T^{4})}{{\left(Var(T)\right)}^{2}}=\frac{\frac{2}{\beta^{\frac{4}{\alpha }}\gamma^{2}}\Gamma \left(\frac{4}{\gamma }\right)\left(2\gamma +4\right)}{{\left(\frac{\left(2\gamma +2\right)\Gamma \left(\frac{2}{\gamma }\right)}{\beta^{\frac{2}{\alpha }}\gamma^{2}}-\frac{{\left(2\gamma +1\right)}^{2}{\left(\Gamma \left(\frac{1}{\gamma }\right)\right)}^{2}}{4\beta^{\frac{2}{\alpha }}\gamma^{4}}\right)}^{2}},$$

$$C.V=\frac{\sqrt{Var(T)}}{E(T)}=\frac{\sqrt{\frac{\left(2\gamma +2\right)\Gamma \left(\frac{2}{\gamma }\right)}{\beta^{\frac{2}{\alpha }}\gamma^{2}}-\frac{{\left(2\gamma +1\right)}^{2}{\left(\Gamma \left(\frac{1}{\gamma }\right)\right)}^{2}}{4\beta^{\frac{2}{\alpha }}\gamma^{4}}}}{\frac{\left(2\gamma +1\right)\Gamma \left(\frac{1}{\gamma }\right)}{2\beta^{\frac{1}{\alpha }}\gamma^{2}}}.$$

The moment-generating function of the PNXL distribution takes the form

$$M(s)=\int_{0}^{\infty }e^{st}f_{PNXL}(t)dt=\sum_{m=0}^{\infty }\frac{s^{m}}{m!}\int_{0}^{\infty }t^{m}f_{PNXL}(t)dt=\sum_{m=0}^{\infty }\frac{s^{m}}{m!}\frac{m\left(m+2\gamma \right)\Gamma \left(\frac{m}{\gamma }\right)}{2\gamma^{2}\beta^{\frac{m}{\alpha }}},$$

its characteristic function is obtained by replacing *s* with *is* in the previous equation.

The $r^{th}$ incomplete moments of PNXL distribution are given by

$$\Psi_{r}(s)=\int_{0}^{s}t^{r}f_{PNXL}(t)dt=\int_{0}^{\infty }t^{r}f_{PNXL}(t)dt-\int_{s}^{\infty }t^{r}f_{PNXL}(t)dt=\frac{r\left(r+2\gamma \right)\Gamma \left(\frac{r}{\gamma }\right)-\gamma^{2}\left(\Gamma (\frac{{}^{r}}{\gamma }+1,s)+\Gamma (\frac{{}^{r}}{\gamma }+2,s)\right)}{2\gamma^{2}\beta^{\frac{r}{\alpha }}},$$

where $\Gamma (\frac{r}{\gamma },s)=\int_{s}^{\infty }t^{\frac{r}{\gamma }-1}e^{-t}dt$.

### 3.4 Stochastic ordering

In this subsection, we consider the stochastic ordering for PNXL distribution. We use the following procedure to obtain remarks about the stochastic ordering.

Consider two random variables $T_{1}$ and $T_{2}$. Then $T_{1}$ is said smaller than $T_{2}$ in the following cases:

1. Stochastic order $(T_{1}{<}_{S}T_{2}$ ), if $F_{T_{1}}(t)<F_{T_{2}}(t)$, ∀*t*.
2. Convex order $\left(T_{1}\leq_{cx}T_{2}\right)$, if for all convex functions *ϕ* and provided expectation exist, $E[\phi (T_{1})]\leq E[\phi (T_{2})]$.
3. Hazard rate order $\left(T_{1}\leq_{hr}T_{2}\right)$, if $h_{T_{1}}(t)\geq h_{T_{2}}(t)$, ∀*t*.
4. Likelihood ratio order $\left(T_{1}{<}_{lr}T_{2}\right)$, if $\frac{f_{T_{1}}(t)}{f_{T_{2}}(t)}$ is decreasing in *t*.

Remark 1 Likelihood ratio order ⇒ hazard rate order ⇒ stochastic order, If $E[T_{1}]=E[T_{2}]$, then convex order ⇔ stochastic order.

Theorem 1 *Let* $T_{i\;}∽PNXLD\left(\beta_{i};\gamma_{i}\right);i=1,2$ *be two random variables. If* $\beta_{1}\geq \beta_{2}$ *and* $\gamma_{1}\leq \gamma_{2}$ *, then* $T_{1}{<}_{lr}T_{2},T_{1}{<}_{hr}T_{2};T_{1}{<}_{S}T_{2}$ *and* $T_{1}\leq_{cx}T_{2}$ *.*

Proof We have

$$\frac{f_{T_{1}}(t)}{f_{T_{2}}\left(t\right)}=\frac{\beta_{1}\gamma_{1}t^{\gamma_{1}-1}\left(\beta_{1}t^{\gamma_{1}}+1\right)}{\beta_{2}\gamma_{2}t^{\gamma_{2}-1}\left(\beta_{2}t^{\gamma_{2}}+1\right)}e^{-\beta_{1}t^{\gamma_{1}}+\beta_{2}t^{\gamma_{2}}}.$$

Using the $\ln\left(\frac{f_{T_{1}}(t)}{f_{T_{2}}\left(t\right)}\right)$ for simplification, we can find

$$\frac{d}{dt}\ln\left(\frac{f_{T_{1}}(t)}{f_{T_{2}}\left(t\right)}\right)=\frac{\beta_{1}\gamma_{1}t^{\gamma_{1}-1}}{\beta_{1}t^{\gamma_{1}}+1}-\frac{\beta_{2}\gamma_{2}t^{\gamma_{2}-1}}{\beta_{2}t^{\gamma_{2}}+1}+\frac{\gamma_{1}-\gamma_{2}}{t}-\beta_{1}\gamma_{1}t^{\gamma_{1}-1}+\beta_{2}\gamma_{2}t^{\gamma_{2}-1},=\frac{\left(\beta_{1}t^{\gamma_{1}}+1\right)\beta_{2}^{2}\gamma_{2}t^{2\gamma_{2}}}{k}-\frac{\left(\beta_{2}t^{\gamma_{2}}+1\right)\beta_{1}^{2}\gamma_{1}t^{2\gamma_{1}}}{k}-\frac{\left(\gamma_{2}-\gamma_{1}\right)\left(\beta_{1}t^{\gamma_{1}}+1\right)\left(\beta_{2}t^{\gamma_{2}}+1\right)}{k},$$

where $k=t\left(\beta_{1}t^{\gamma_{1}}+1\right)\left(\beta_{2}t^{\gamma_{2}}+1\right)$. To this end, if $\beta_{1}\geq \beta_{2}$ and $\gamma_{1}\leq \gamma_{2}$, we have $\frac{d}{dt}\ln\left(\frac{f_{T_{1}}(t)}{f_{T_{2}}\left(t\right)}\right)\leq 0$. This means that $T_{1}{<}_{lr}T_{2}$. Also, the theorem is proved according to Remark 1. □

## 4 Fuzzy reliability

In this section, we present the fuzzy reliability of PNXL distribution.

Let *T* be a continuous random variable representing a system's failure time (component). The fuzzy reliability can be calculated using the fuzzy probability formula as follows:

$$R_{Fuzzy}(t)=P\left(T>t\right)=\int_{t}^{\infty }\nu (y)\;f_{PNXL}(y)dy,0\leq t\leq y<\infty ,$$

where ν(y) is a membership function that describes the degree to which each element of a given universe belongs to a fuzzy set. Now, assume that ν(y) is

(3) $$\nu (y)=\left\{ \begin{array}{cc} 0\; & ,y\leq t_{1}\\ \frac{y-t_{1}}{t_{2}-t_{1}}\; & ,t_{1}<y<t_{2,}t_{1}\geq 0\\ 1\; & ,y\geq t_{2} \end{array}\right.$$

For ν(y), by the computational analysis of the function of fuzzy numbers, the lifetime y(δ) can be obtained corresponds to a certain value of δ−Cut,δ∈[0,1], can be obtained as: $\nu (y)=\delta \to \;\frac{y-t_{1}}{t_{2}-t_{1}}=\delta$, then

$$\left\{ \begin{array}{cc} y(\delta )\leq t_{1}\; & ,\delta =0\\ y(\delta )=t_{1}+\delta (t_{2}-t_{1})\; & ,0<\delta <1\\ y(\delta )\geq t_{2}\; & ,\delta =1 \end{array}\right.$$

As a result, the fuzzy reliability values may be determined for all *δ* values. The fuzzy dependability of the PNXL distribution is determined by the fuzzy reliability definition. The fuzzy reliability of the PNXL distribution can be defined as,

$$R_{Fuzzy}(t)=\left(\frac{1}{2}\beta t_{1}^{\gamma }+1\right)e^{-\beta t_{1}^{\gamma }}-\left(\frac{1}{2}\beta y{\left(\delta \right)}^{\gamma }+1\right)e^{-\beta y{\left(\delta \right)}^{\gamma }}.$$

Then $R_{Fuzzy}{\left(t\right)}_{\gamma =0}=0$.

## 5 Actuarial measures

In this section, some actuarial measures of the PNXL distribution, such as mean excess function, limited expected value function, tail value at risk, and tail variance.

### 5.1 Mean excess function

For a claim amount random variable *T*, the mean excess function or mean residual life function is the expected payment per claim on a policy with a fixed amount deductible of *t*, where claims with amounts less than or equal to *t* are completely ignored. It is defined for the PNXL distribution as follows

$$e(t)=E(T-t/T>t)=\frac{1}{S\left(t\right)}\int_{t}^{\infty }S\left(u\right)du=\frac{1}{\left(\frac{1}{2}\beta t^{\gamma }+1\right)e^{-\beta t^{\gamma }}}\int_{t}^{\infty }\left(\frac{1}{2}\beta u^{\gamma }+1\right)e^{-\beta u^{\gamma }}du=\frac{\Gamma \left(\left(1+\frac{1}{\gamma }\right),\beta t^{\gamma }\right)+2\Gamma \left(\frac{1}{\gamma },\beta t^{\gamma }\right)}{2\beta^{\frac{1}{\gamma }}\gamma \left(\frac{1}{2}\beta t^{\gamma }+1\right)e^{-\beta t^{\gamma }}}.$$

### 5.2 Limited expected value function

The limited expected value function *L* of a claim size variable *T* or of the corresponding c.d.f. F(t), is defined as follows

$$L(t)=E\{\min(T,t)\}=\int_{0}^{t}udF(u)+tS(t),\;t>0.$$

The value of the function *L* at point *t* is equal to the expectation of the c.d.f. F(t) truncated at this point. Given a policy limit or deductible from a reinsurance perspective, say *t*, a limited loss random variable is defined as follows

$$T\wedge t=\min(T,t)=\left\{ \begin{array}{cc} T,\; & T\leq t\\ t,\; & T>t \end{array}\right..$$

The limited expected value function is defined as the expectation of the limited, which is calculated as follows

$$E(T\wedge t)=\int_{0}^{t}uf(u)du+tS(t)=m_{1}(t)+tS(t),$$

where

$$m_{1}(t)=\int_{0}^{t}uf(u)du=\frac{1}{2\beta^{\frac{1}{\gamma }}}\left(\Gamma \left(\left(2+\frac{1}{\gamma }\right)\right)+\Gamma \left(\left(1+\frac{1}{\gamma }\right)\right)\right)-\frac{1}{2}\frac{\beta }{\beta^{\frac{1}{\gamma }+1}}\Gamma \left(\frac{1}{\gamma }+2,t\beta \right)-\frac{1}{2\beta^{\frac{1}{\gamma }}}\Gamma \left(\frac{1}{\gamma }\left(\gamma +1\right),t\beta \right).$$

Then, we have

$$E(T\wedge t)=\frac{1}{2\beta^{\frac{1}{\gamma }}}\left(\Gamma \left(\left(2+\frac{1}{\gamma }\right)\right)+\Gamma \left(\left(1+\frac{1}{\gamma }\right)\right)\right)-\frac{1}{2}\frac{\beta }{\beta^{\frac{1}{\gamma }+1}}\Gamma \left(\frac{1}{\gamma }+2,t\beta \right)-\frac{1}{2\beta^{\frac{1}{\gamma }}}\Gamma \left(\frac{1}{\gamma }\left(\gamma +1\right),t\beta \right)+t\left(\frac{1}{2}\beta t^{\gamma }+1\right)e^{-\beta t^{\gamma }}.$$

### 5.3 Tail value at risk

The tail value at risk (TVaR), also known as the tail conditional expectation is a risk measure associated with the general value at risk. TVaR measures the expectation of the losses beyond VaR. The TVaR is defined for the PNXL distribution as follows

$$TVaR=E(T|T>VaR)=\frac{1}{1-p}\int_{VaR}^{\infty }tf(t)dt=\frac{1}{2(1-p)\beta^{\frac{1}{\gamma }}}\left[\Gamma \left(\frac{1}{\gamma }+2,{\left(VaR\right)}^{\gamma }\beta \right)+\Gamma \left(1+\frac{1}{\gamma },{\left(VaR\right)}^{\gamma }\beta \right)\right].$$

Although it virtually always represents a loss, *VaR* is conventionally reported as a positive number.

### 5.4 Tail variance

Tail variance (*TV*) measures losses' conditional variance, given that they exceed VaR at a given probability *P*. *TV* is defined for the PNXL distribution as follows

$$TV=E(T^{2}|T>VaR)-{\left(TVaR\right)}^{2}=\frac{1}{1-p}\int_{VaR}^{\infty }t^{2}f(t)dt-{\left(TVaR\right)}^{2}=\frac{1}{2\left(1-p\right)\beta^{\frac{2}{\gamma }}}\left[\Gamma \left(\frac{2}{\gamma }+2,\beta {\left(VaR\right)}^{\gamma }\right)+\Gamma \left(1+\frac{2}{\gamma },\beta {\left(VaR\right)}^{\gamma }\right)\right]-{\left[\frac{1}{2(1-p)\beta^{\frac{1}{\gamma }}}\left(\Gamma \left(\frac{1}{\gamma }+2,{\left(VaR\right)}^{\gamma }\beta \right)+\Gamma \left(1+\frac{1}{\gamma },{\left(VaR\right)}^{\gamma }\beta \right)\right)\right]}^{2}.$$

## 6 Estimation methods

In this section, we discuss sixteen estimation methods namely, maximum likelihood, Anderson Darling, Cramer-von Mises, maximum product spacings, least squares, percentile, right tail Anderson Darling, right tail Anderson-Darling, weighted least squares, left tail Anderson Darling, minimum spacing absolute distance, minimum spacing absolute log-distance, Anderson Darling left tail second order, Kolmogorov, minimum spacing square distance, minimum spacing square log-distance, and minimum spacing Linex distance methods.

### 6.1 Maximum likelihood method

In this subsection, we present the maximum likelihood method to estimate the parameters of the PXNL distribution. The log-likelihood function is

(4) $$\ell \left(\beta ,\gamma |x_{i}\right)=\log\left(\frac{\beta \gamma }{2}\right)+\sum_{i=1}^{n}\log\left(\beta \gamma x_{i}^{\gamma -1}\left(\beta x_{i}^{\gamma }+1\right)\right)-\beta \sum_{i=1}^{n}x_{i}^{\gamma }.$$

where $x_{i}=\left(x_{1},x_{2},...,x_{n}\right)$ for i=1,2,...n. The maximum likelihood estimators (MLEs) can be derived by maximizing the log-likelihood function in Eq. (4).

### 6.2 Method of Anderson-Darling

The Anderson Darling method is proposed by [3]. This method is used to estimate the parameters of PNXL distribution. Let $x_{i:n}=x_{1:n},...,x_{n:n}$ be an ordered sample of values from the PNXL distribution. The Anderson Darling estimators (ADEs) are obtained by minimizing the function given in Eq. (5).

(5) $$A(x_{i})=-n-\frac{1}{n}\sum_{i=1}^{n}(2i-1)\left[\log F(x_{i:n})+\log S(x_{n-i-1:n})\right].$$

### 6.3 Method of Cramer-von Mises

In this subsection, Cramer-von Mises, one of the popular estimation methods for estimating the parameters of the PNXL distribution, is analyzed. The Cramer-von Mises method is introduced by [6]. The Cramer-von Mises estimators (CVMEs) are obtained by minimizing the function in Eq. (6).

(6) $$C(x_{i})=\frac{1}{12n}+\sum_{i=1}^{n}{\left[F(x_{i:n})-\frac{2i-1}{2n}\right]}^{2}$$

### 6.4 Method of maximum product of spacings

This subsection provides the maximum product spacing method for the point estimation of the PNXL distribution. The maximum product spacings method was suggested by Cheng and Amin [5] as an alternative to the maximum likelihood method.

The maximum product spacings estimators (MPSEs) are derived by maximizing the function given in Eq. (7)

(7) $$\delta \left(x_{i}\right)=\frac{1}{n+1}\sum_{i=1}^{n+1}\log\Lambda_{i}(x_{i}),$$

where $\Lambda_{i}(x_{i})=F(x_{i:n})-F(x_{i-1:n})$, $F(x_{0:n})=0$ and $F(x_{n+1:n})=1$.

### 6.5 Method of least squares

The least squares method is suggested by [23]. This method of the least squares is commonly used for point estimation of various lifetime distributions. In this regard, we consider the least squares method to estimate the parameters of the PNXL distribution. The least squares estimators (OLSEs) are obtained by minimizing the function given in Eq. (8).

(8) $$V(x_{i})=\sum_{i=1}^{n}{\left[F(x_{i:n})-\frac{i}{n+1}\right]}^{2}.$$

### 6.6 Method of percentile

The percentile estimation method is proposed by Kao [9] and [10]. The percentile estimators (PCEs) are obtained by minimizing the function given in Eq. (9) concerning *β* and *γ* parameters.

(9) $$PCE=\sum_{i=1}^{n}{\left[x_{i:n}-Q(p_{i})\right]}^{2},\;p_{i}=\frac{i}{n+1}.$$

### 6.7 Method of right tail Anderson darling

This subsection presents the right tail Anderson Darling estimators (RTADEs) of the parameters of the PNXL distribution. The RTADEs are derived by minimizing the function in Eq. (10) concerning *β* and *γ* parameters.

(10) $$R(x_{i})=\frac{n}{2}-2\sum_{i=1}^{n}F(x_{i:n})-\frac{1}{n}\sum_{i=1}^{n}(2i-1)\log S(x_{i:n}).$$

### 6.8 Method of weighted least squares

The weighted least squares method is suggested by [23]. The weighted least squares estimators (WLSEs) are derived by minimizing the function in Eq. (11).

(11) $$W(x_{i})=\sum_{i=1}^{n}\frac{{\left(n+1\right)}^{2}(n+2)}{i(n-i+1)}{\left[F(x_{i:n})-\frac{i}{n+1}\right]}^{2}.$$

### 6.9 Method of left tail Anderson darling

In this subsection, we discuss the left tail Anderson Darling estimators (LTADEs) of the parameters of PNXL distribution. LTADEs are obtained by minimizing the function in Eq. (12) concerning *β* and *γ* parameters.

(12) $$L(x_{i})=-\frac{3}{2}n+2\sum_{i=1}^{n}F(x_{i:n})-\frac{1}{n}\sum_{i=1}^{n}(2i-1)\log F(x_{i:n}).$$

### 6.10 Method of minimum spacing absolute distance

Method of minimum spacing absolute distance is studied by [9]. We examine the minimum spacing absolute distance estimators (MSADEs) of the parameters of PNXL distribution. The MSADEs are obtained by minimizing the function in Eq. (13) concerning *β* and *γ* parameters.

(13) $$\zeta \left(x_{i}\right)=\sum_{i=1}^{n+1}|\Lambda_{i}-\frac{1}{n+1}|.$$

### 6.11 Method of minimum spacing absolute-log distance

The method of minimum spacing absolute log distance is introduced by [24]. The minimum spacing absolute log distance estimators (MSALDEs) are derived by minimizing the function in Eq. (14)

(14) $$ϒ\left(x_{i}\right)=\sum_{i=1}^{n+1}|\log\Lambda_{i}-\log\frac{1}{n+1}|.$$

### 6.12 Anderson darling left tail second order

In this subsection, we present the method of Anderson Darling's left tail second order for the PNXL distribution. For more details, see [1]. Anderson Darling left tail second order estimators (ADSOEs) are derived by minimizing the function in Eq. (15).

(15) $$LTS=2\sum_{i=1}^{n}\log F(x_{i})+\frac{1}{n}\sum_{i=1}^{n}\frac{\left(2i-1\right)}{F(x_{i})}.$$

### 6.13 Kolmogorov method

In this subsection, we discuss one of the estimation methods, the Kolmogorov method to estimate the parameters of PNXL distribution. For more details about the Kolmogorov method, see [1]. Kolmogorov estimators (KEs) are derived by minimizing the function in Eq. (16).

(16) $$KM=\underset{1\leq i\leq n}{MAX}\left[\frac{i}{n}-F(x_{i}),F(x_{i})-\frac{i-1}{n}\right].$$

### 6.14 Method of the minimum spacing square distance

We consider another estimation method, which is the minimum spacing square distance. For more details about this method, see [24]. The minimum spacing distance estimators (MSSDs) can be obtained by minimizing the function in Eq. (17).

(17) $$\phi_{\left(x_{i}\right)}=\sum_{i=1}^{n+1}{\left(I_{i}-\frac{1}{n+1}\right)}^{2}.$$

### 6.15 Method of the minimum spacing square-log distance

In this subsection, similarly Section 6.14, we focus on the minimum spacing square log distance. For more details about this method, see [24]. The minimum spacing log distance estimators (MSSLDs) are derived by minimizing the function in Eq. (18).

(18) $$\Psi_{\left(x_{i}\right)}=\sum_{i=1}^{n+1}{\left(\log I_{i}-\log\frac{1}{n+1}\right)}^{2}.$$

We notice that the function given in Eq. (18) is the logarithm of the function in Eq. (17). Therefore, this method is named the minimum spacing square-log distance.

### 6.16 Method of the minimum spacing linex distance

Lastly, we studied the minimum spacing Linex distance method to estimate the PNXL distribution parameters in Section 6. For more details about this estimator, see [24]. The minimum spacing Linex distance estimators (MSLDs) are obtained by minimizing the function in Eq. (19).

(19) $$\Delta_{\left(x_{i}\right)}=\sum_{i=1}^{n+1}\left[e^{I_{i}-\frac{1}{n+1}}-\left(I_{i}-\frac{1}{n+1}\right)-1\right].$$

## 7 Numerical simulation

**Figure 1:**
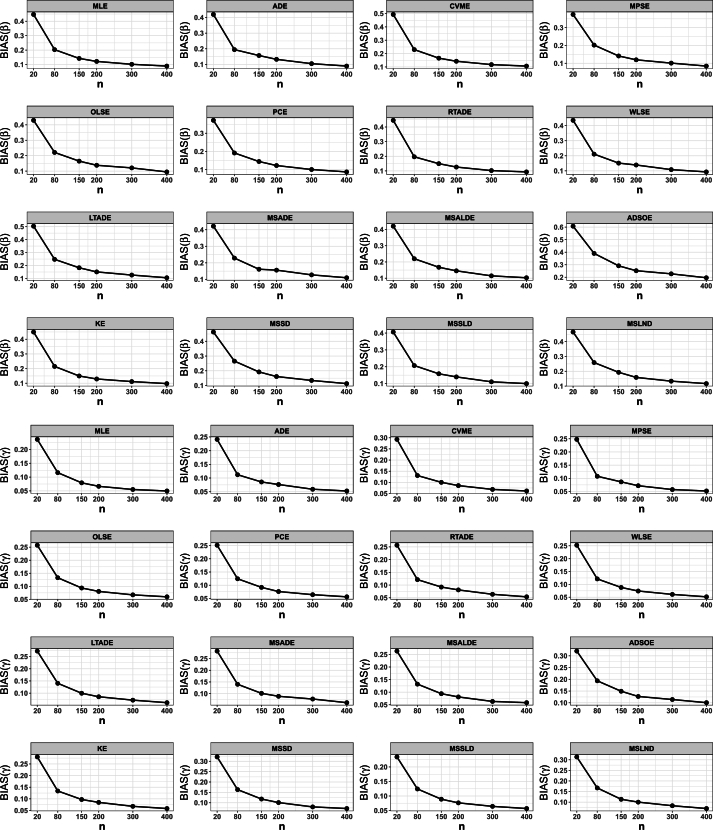
Graphical representation for BIAS values presented in Table 1.

**Figure 2:**
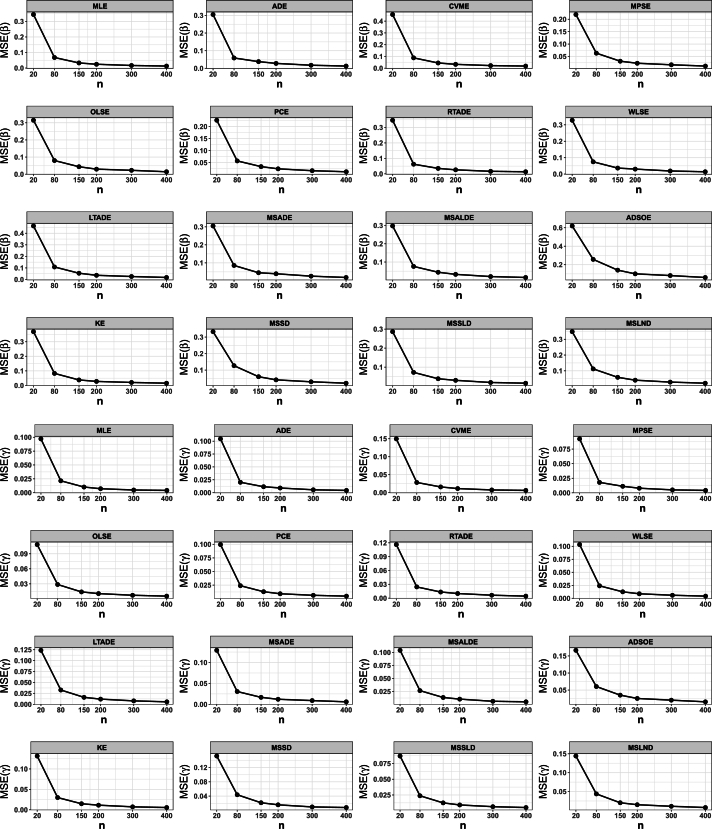
Graphical representation for MSE values presented in Table 1.

**Figure 3:**
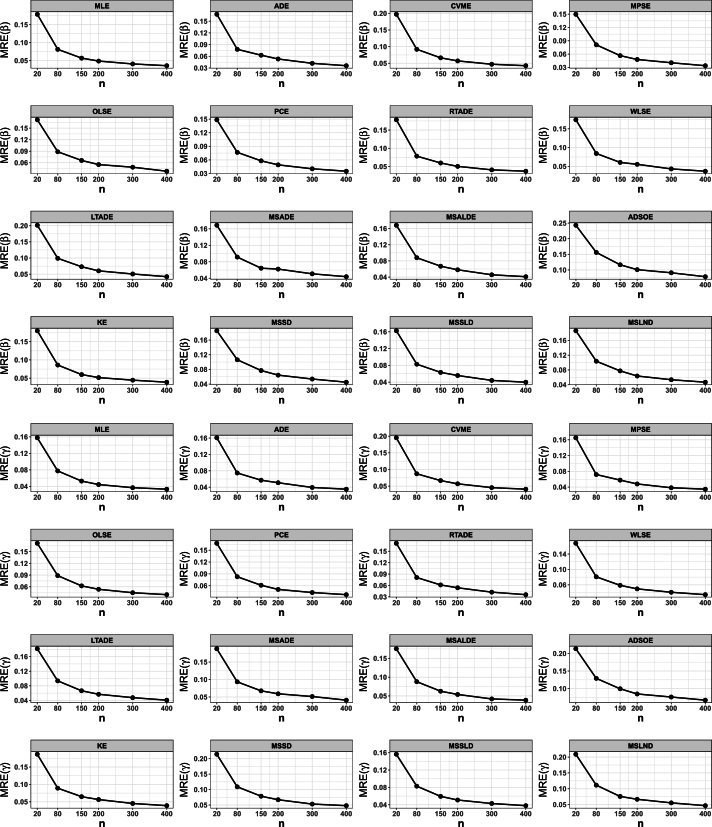
Graphical representation for MRE values presented in Table 1.

**Figure 4:**
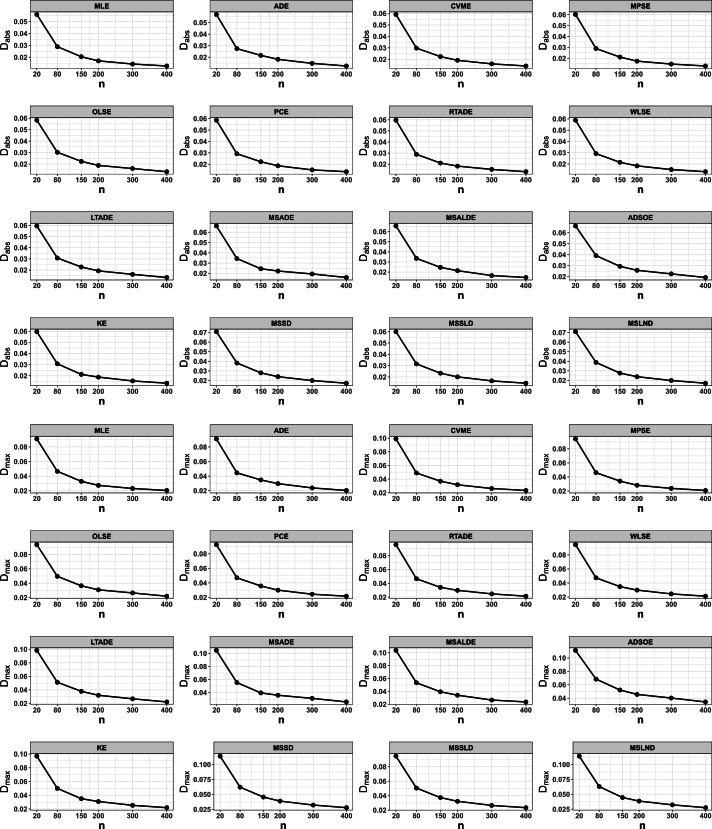
Graphical representation for *D*_*abs*_ and *D*_*max*_ values presented in Table 1.

**Figure 5:**
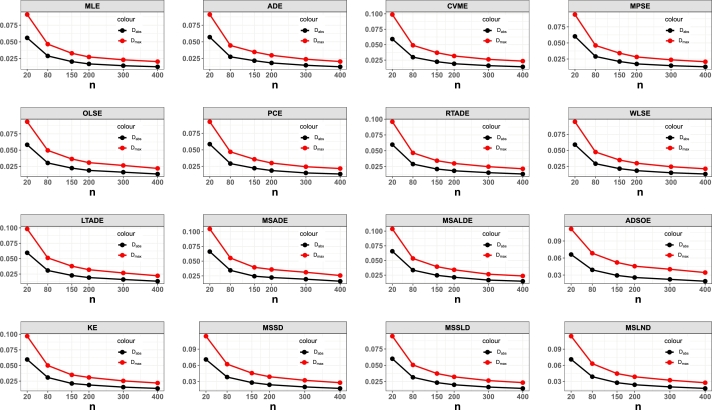
Comparison between *D*_*abs*_ and *D*_*max*_ values presented in Table 1.

**Figure 6:**
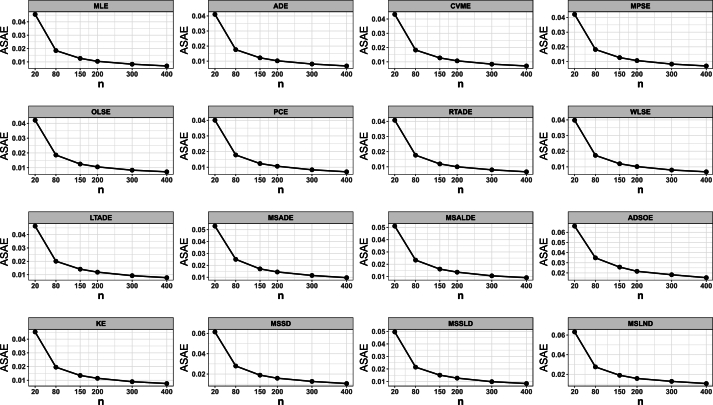
Graphical representation for ASAE values presented in Table 1.
• It is noted that the behavior of all calculated measures for all estimation methods decreases as sample size increases.
• In small-size samples, PCE and ADE outperform MLE according to the MSE criterion. However, as the sample size increases, the performance of the MLE improves and outperforms its competitors. In large sample sizes, MPSE is the estimator that performs closest to MLE for β=2.5,γ=1.5.
• The KE is the best estimator according to MSE criteria for β=0.5,γ=0.3.
• In small-size samples, MPSE outperforms MLE according to the MSE criterion. However, as the sample size increases, the performance of the MLE improves and outperforms its competitors for β=2,γ=0.7.
• According to the MSE criterion, MPSE is the best estimator in n=150 and n=400, while MLE is the best estimator in other cases for β=0.8,γ=0.2.
• According to the MSE criterion, MLE is the best estimator in n=200 and n=400, while PCE is the best estimator in other cases for β=4,γ=2.5.
• Table 6 provides overall ranks of all estimation methods. From Table 6, we conclude that the MLE is the best estimation method for our model.

In this section, we provide an extensive Monte Carlo simulation study to assess the performances of the mentioned estimators in Section 6. The details of the simulation study are listed as follows:

• The repetition number in the simulation study is one thousand times.
• Sample sizes n=20,80,150,200,200,300,400.
• Five different parameter settings were used as follows: β=2.5,γ=1.5; β=0.5,γ=0.3; β=2,γ=0.7; β=0.9,γ=0.2; β=4,γ=2.5.
• Six different measures are used for comparison between different estimation methods, such as bias (BIAS), mean squared errors (MSE), mean absolute relative errors (MRE), average absolute difference ($D_{abs}$), maximum absolute difference ($D_{max}$), and average squared absolute error (ASAE).
• Numerical results are written in Table 1, Table 2, Table 3, Table 4, Table 5 and a graphical representation of Table 1 in Figure 1, Figure 2, Figure 3, Figure 4, Figure 5, Figure 6.

**Table 1:** Numerical values of simulation measures for *β* = 2.5, *γ* = 1.5.

n	Est.	MLE	ADE	CVME	MPSE	OLSE	PCE	RTADE	WLSE	LTADE	MSADE	MSALDE	ADSOE	KE	MSSD	MSSLD	MSLND
20	BIAS($\overset{ˆ}{\beta }$)	0.44703^{10}^	0.41946^{4}^	0.49297^{14}^	0.37424^{2}^	0.42935^{7}^	0.37136^{1}^	0.44558^{9}^	0.43507^{8}^	0.50335^{15}^	0.42147^{6}^	0.42014^{5}^	0.60756^{16}^	0.44958^{11}^	0.46337^{12}^	0.40627^{3}^	0.46524^{13}^
	BIAS($\overset{ˆ}{\gamma }$)	0.23669^{2}^	0.24115^{3}^	0.29284^{13}^	0.24781^{4}^	0.25744^{8}^	0.25141^{5}^	0.25664^{7}^	0.25225^{6}^	0.2725^{10}^	0.28206^{12}^	0.26337^{9}^	0.32025^{15}^	0.28022^{11}^	0.32144^{16}^	0.23452^{1}^	0.31355^{14}^
	MSE($\overset{ˆ}{\beta }$)	0.34424^{10}^	0.30502^{5}^	0.4561^{14}^	0.21916^{1}^	0.31481^{7}^	0.22629^{2}^	0.34658^{11}^	0.32808^{8}^	0.4633^{15}^	0.30505^{6}^	0.29755^{4}^	0.61949^{16}^	0.36885^{13}^	0.33288^{9}^	0.28663^{3}^	0.34849^{12}^
	MSE($\overset{ˆ}{\gamma }$)	0.09708^{3}^	0.10446^{7}^	0.14946^{14}^	0.09254^{2}^	0.10832^{8}^	0.09947^{4}^	0.11613^{9}^	0.10332^{5}^	0.12363^{10}^	0.12921^{11}^	0.10406^{6}^	0.16655^{16}^	0.13171^{12}^	0.15211^{15}^	0.08693^{1}^	0.14406^{13}^
	MRE($\overset{ˆ}{\beta }$)	0.17881^{10}^	0.16778^{4}^	0.19719^{14}^	0.14969^{2}^	0.17174^{7}^	0.14854^{1}^	0.17823^{9}^	0.17403^{8}^	0.20134^{15}^	0.16859^{6}^	0.16806^{5}^	0.24302^{16}^	0.17983^{11}^	0.18535^{12}^	0.16251^{3}^	0.1861^{13}^
	MRE($\overset{ˆ}{\gamma }$)	0.15779^{2}^	0.16077^{3}^	0.19523^{13}^	0.16521^{4}^	0.17163^{8}^	0.16761^{5}^	0.17109^{7}^	0.16817^{6}^	0.18167^{10}^	0.18804^{12}^	0.17558^{9}^	0.2135^{15}^	0.18681^{11}^	0.21429^{16}^	0.15634^{1}^	0.20903^{14}^
	*D*_*abs*_	0.056^{1}^	0.05706^{2}^	0.05911^{6}^	0.06021^{11}^	0.05824^{3}^	0.05865^{4}^	0.05968^{9}^	0.05886^{5}^	0.05953^{7}^	0.06621^{13}^	0.06568^{12}^	0.06622^{14}^	0.05959^{8}^	0.07085^{15}^	0.06001^{10}^	0.07096^{16}^
	*D*_*max*_	0.09098^{1}^	0.09111^{2}^	0.09887^{11}^	0.09414^{5}^	0.09363^{4}^	0.09255^{3}^	0.09593^{8}^	0.0946^{6}^	0.09831^{10}^	0.10463^{13}^	0.10372^{12}^	0.11126^{14}^	0.09693^{9}^	0.11404^{15}^	0.09466^{7}^	0.11412^{16}^
	ASAE	0.04559^{1}^	0.04104^{2}^	0.04326^{11}^	0.04212^{5}^	0.04211^{4}^	0.04004^{3}^	0.04077^{8}^	0.0398^{6}^	0.04638^{10}^	0.05298^{13}^	0.05105^{12}^	0.06617^{14}^	0.04526^{9}^	0.06136^{15}^	0.04956^{7}^	0.06316^{16}^
	∑*Ranks*	48^{5}^	34^{2}^	106^{13}^	37^{3}^	57^{7}^	27^{1}^	72^{8}^	53^{6}^	102^{12}^	92^{10}^	74^{9}^	138^{16}^	94^{11}^	124^{14}^	40^{4}^	126^{15}^
80	BIAS($\overset{ˆ}{\beta }$)	0.20328^{5}^	0.19463^{2}^	0.23^{12}^	0.20228^{4}^	0.22114^{10}^	0.19175^{1}^	0.19635^{3}^	0.21089^{7}^	0.24768^{13}^	0.22866^{11}^	0.21979^{9}^	0.39034^{16}^	0.215^{8}^	0.26575^{15}^	0.20703^{6}^	0.25907^{14}^
	BIAS($\overset{ˆ}{\gamma }$)	0.11609^{3}^	0.11204^{2}^	0.13059^{8}^	0.10776^{1}^	0.13292^{10}^	0.12432^{7}^	0.12146^{5}^	0.12129^{4}^	0.14046^{13}^	0.14008^{12}^	0.13201^{9}^	0.19366^{16}^	0.13389^{11}^	0.16329^{14}^	0.12428^{6}^	0.16707^{15}^
	MSE($\overset{ˆ}{\beta }$)	0.0676^{5}^	0.05865^{2}^	0.08795^{12}^	0.06322^{4}^	0.0799^{9}^	0.05717^{1}^	0.06247^{3}^	0.07448^{7}^	0.10839^{13}^	0.0855^{11}^	0.07615^{8}^	0.25727^{16}^	0.08279^{10}^	0.1267^{15}^	0.07247^{6}^	0.11232^{14}^
	MSE($\overset{ˆ}{\gamma }$)	0.02142^{3}^	0.02015^{2}^	0.02823^{9}^	0.01793^{1}^	0.02874^{10}^	0.02394^{5}^	0.02443^{7}^	0.02405^{6}^	0.03278^{13}^	0.0307^{12}^	0.02664^{8}^	0.06037^{16}^	0.02998^{11}^	0.04376^{15}^	0.02374^{4}^	0.0437^{14}^
	MRE($\overset{ˆ}{\beta }$)	0.08131^{5}^	0.07785^{2}^	0.092^{12}^	0.08091^{4}^	0.08846^{10}^	0.0767^{1}^	0.07854^{3}^	0.08436^{7}^	0.09907^{13}^	0.09146^{11}^	0.08792^{9}^	0.15614^{16}^	0.086^{8}^	0.1063^{15}^	0.08281^{6}^	0.10363^{14}^
	MRE($\overset{ˆ}{\gamma }$)	0.07739^{3}^	0.07469^{2}^	0.08706^{8}^	0.07184^{1}^	0.08861^{10}^	0.08288^{7}^	0.08097^{5}^	0.08086^{4}^	0.09364^{13}^	0.09339^{12}^	0.08801^{9}^	0.12911^{16}^	0.08926^{11}^	0.10886^{14}^	0.08285^{6}^	0.11138^{15}^
	*D*_*abs*_	0.02892^{3}^	0.02744^{1}^	0.02979^{7}^	0.02895^{4}^	0.03027^{8}^	0.02921^{6}^	0.02887^{2}^	0.0292^{5}^	0.03078^{10}^	0.03443^{13}^	0.03356^{12}^	0.03914^{16}^	0.03077^{9}^	0.03825^{14}^	0.03155^{11}^	0.03876^{15}^
	*D*_*max*_	0.04661^{4}^	0.04452^{1}^	0.04902^{7}^	0.0461^{2}^	0.04958^{8}^	0.04701^{5}^	0.04659^{3}^	0.04738^{6}^	0.05122^{11}^	0.05534^{13}^	0.05342^{12}^	0.06849^{16}^	0.05003^{9}^	0.06216^{14}^	0.05049^{10}^	0.06302^{15}^
	ASAE	0.01849^{4}^	0.01763^{1}^	0.01828^{7}^	0.01821^{2}^	0.01858^{8}^	0.01782^{5}^	0.01768^{3}^	0.01735^{6}^	0.02008^{11}^	0.02505^{13}^	0.02337^{12}^	0.03483^{16}^	0.01956^{9}^	0.0279^{14}^	0.02147^{10}^	0.02758^{15}^
	∑*Ranks*	38^{5}^	16^{1}^	81^{8}^	26^{2}^	83^{9}^	37^{4}^	34^{3}^	47^{6}^	109^{13}^	108^{12}^	88^{11}^	144^{16}^	86^{10}^	131^{15}^	66^{7}^	130^{14}^
150	BIAS($\overset{ˆ}{\beta }$)	0.14234^{2}^	0.15693^{7}^	0.16586^{11}^	0.14125^{1}^	0.16479^{10}^	0.14483^{3}^	0.14947^{4}^	0.15137^{6}^	0.18256^{13}^	0.16127^{9}^	0.16743^{12}^	0.29144^{16}^	0.14964^{5}^	0.19229^{14}^	0.15817^{8}^	0.1934^{15}^
	BIAS($\overset{ˆ}{\gamma }$)	0.07923^{1}^	0.0856^{2}^	0.10005^{12}^	0.08652^{3}^	0.09363^{8}^	0.09181^{6}^	0.09223^{7}^	0.08808^{4}^	0.0997^{11}^	0.10136^{13}^	0.09396^{9}^	0.14924^{16}^	0.09721^{10}^	0.11779^{15}^	0.08904^{5}^	0.11353^{14}^
	MSE($\overset{ˆ}{\beta }$)	0.03264^{2}^	0.03794^{7}^	0.0444^{11}^	0.03088^{1}^	0.04381^{9}^	0.03342^{3}^	0.03529^{4}^	0.03635^{5}^	0.05419^{13}^	0.045^{12}^	0.0443^{10}^	0.14072^{16}^	0.03783^{6}^	0.05993^{15}^	0.03884^{8}^	0.05892^{14}^
	MSE($\overset{ˆ}{\gamma }$)	0.01016^{1}^	0.01149^{3}^	0.01579^{11}^	0.01125^{2}^	0.01394^{9}^	0.01293^{6}^	0.01337^{7}^	0.0125^{5}^	0.016^{12}^	0.01677^{13}^	0.0135^{8}^	0.03519^{16}^	0.01483^{10}^	0.02141^{15}^	0.01244^{4}^	0.02025^{14}^
	MRE($\overset{ˆ}{\beta }$)	0.05694^{2}^	0.06277^{7}^	0.06634^{11}^	0.0565^{1}^	0.06592^{10}^	0.05793^{3}^	0.05979^{4}^	0.06055^{6}^	0.07303^{13}^	0.06451^{9}^	0.06697^{12}^	0.11658^{16}^	0.05985^{5}^	0.07691^{14}^	0.06327^{8}^	0.07736^{15}^
	MRE($\overset{ˆ}{\gamma }$)	0.05282^{1}^	0.05706^{2}^	0.0667^{12}^	0.05768^{3}^	0.06242^{8}^	0.06121^{6}^	0.06149^{7}^	0.05872^{4}^	0.06647^{11}^	0.06757^{13}^	0.06264^{9}^	0.0995^{16}^	0.06481^{10}^	0.07853^{15}^	0.05936^{5}^	0.07568^{14}^
	*D*_*abs*_	0.02045^{1}^	0.02159^{6}^	0.02241^{9}^	0.02112^{3}^	0.02236^{8}^	0.02222^{7}^	0.02099^{2}^	0.02147^{5}^	0.02273^{10}^	0.02446^{12}^	0.02469^{13}^	0.02934^{16}^	0.02123^{4}^	0.02801^{15}^	0.02337^{11}^	0.02759^{14}^
	*D*_*max*_	0.03292^{1}^	0.03475^{4}^	0.03701^{9}^	0.0341^{2}^	0.03639^{8}^	0.03576^{7}^	0.03421^{3}^	0.03486^{5}^	0.03782^{11}^	0.0395^{13}^	0.03944^{12}^	0.05221^{16}^	0.03497^{6}^	0.04558^{15}^	0.03733^{10}^	0.04457^{14}^
	ASAE	0.01262^{1}^	0.01219^{4}^	0.01276^{9}^	0.01267^{2}^	0.0125^{8}^	0.01233^{7}^	0.01191^{3}^	0.01198^{5}^	0.01413^{11}^	0.01701^{13}^	0.01605^{12}^	0.02572^{16}^	0.01349^{6}^	0.01892^{15}^	0.01507^{10}^	0.01906^{14}^
	∑*Ranks*	17^{1}^	41^{4}^	94^{10}^	23^{2}^	75^{9}^	45^{6}^	39^{3}^	42^{5}^	104^{12}^	107^{13}^	97^{11}^	144^{16}^	65^{7}^	132^{15}^	70^{8}^	129^{14}^
200	BIAS($\overset{ˆ}{\beta }$)	0.12174^{2}^	0.13256^{6}^	0.14279^{10}^	0.11962^{1}^	0.13783^{7}^	0.12273^{3}^	0.12625^{4}^	0.13881^{8}^	0.15071^{12}^	0.15591^{13}^	0.14527^{11}^	0.25232^{16}^	0.1291^{5}^	0.16102^{15}^	0.13951^{9}^	0.15896^{14}^
	BIAS($\overset{ˆ}{\gamma }$)	0.0666^{1}^	0.07629^{5}^	0.08545^{12}^	0.07185^{2}^	0.08056^{7}^	0.07604^{4}^	0.08098^{8}^	0.07418^{3}^	0.08529^{11}^	0.08875^{13}^	0.081^{9}^	0.12703^{16}^	0.08483^{10}^	0.10057^{15}^	0.0764^{6}^	0.10011^{14}^
	MSE($\overset{ˆ}{\beta }$)	0.02421^{2}^	0.02793^{6}^	0.03285^{11}^	0.02256^{1}^	0.02972^{7}^	0.02424^{3}^	0.02601^{4}^	0.03019^{8}^	0.03627^{12}^	0.03937^{13}^	0.03277^{10}^	0.10057^{16}^	0.02791^{5}^	0.04087^{15}^	0.03059^{9}^	0.04065^{14}^
	MSE($\overset{ˆ}{\gamma }$)	0.00712^{1}^	0.00902^{4}^	0.01129^{10}^	0.00796^{2}^	0.01058^{9}^	0.00915^{6}^	0.01012^{7}^	0.00872^{3}^	0.01193^{12}^	0.01218^{13}^	0.01034^{8}^	0.02547^{16}^	0.01136^{11}^	0.01559^{15}^	0.00914^{5}^	0.01507^{14}^
	MRE($\overset{ˆ}{\beta }$)	0.0487^{2}^	0.05302^{6}^	0.05711^{10}^	0.04785^{1}^	0.05513^{7}^	0.04909^{3}^	0.0505^{4}^	0.05552^{8}^	0.06029^{12}^	0.06236^{13}^	0.05811^{11}^	0.10093^{16}^	0.05164^{5}^	0.06441^{15}^	0.05581^{9}^	0.06358^{14}^
	MRE($\overset{ˆ}{\gamma }$)	0.0444^{1}^	0.05086^{5}^	0.05697^{12}^	0.0479^{2}^	0.0537^{7}^	0.05069^{4}^	0.05398^{8}^	0.04945^{3}^	0.05686^{11}^	0.05917^{13}^	0.054^{9}^	0.08469^{16}^	0.05655^{10}^	0.06705^{15}^	0.05093^{6}^	0.06674^{14}^
	*D*_*abs*_	0.01697^{1}^	0.0182^{3}^	0.01917^{9}^	0.01739^{2}^	0.01883^{8}^	0.01867^{6}^	0.01823^{4}^	0.01834^{5}^	0.01924^{10}^	0.02226^{13}^	0.02139^{12}^	0.02571^{16}^	0.01881^{7}^	0.02401^{15}^	0.02008^{11}^	0.0238^{14}^
	*D*_*max*_	0.02739^{1}^	0.02968^{3}^	0.03172^{9}^	0.02819^{2}^	0.03077^{7}^	0.03003^{6}^	0.02988^{4}^	0.0299^{5}^	0.03199^{10}^	0.03588^{13}^	0.03412^{12}^	0.04564^{16}^	0.03088^{8}^	0.03886^{15}^	0.03224^{11}^	0.03862^{14}^
	ASAE	0.01045^{1}^	0.0102^{3}^	0.0107^{9}^	0.01064^{2}^	0.01056^{7}^	0.01062^{6}^	0.01001^{4}^	0.01018^{5}^	0.0119^{10}^	0.01442^{13}^	0.01354^{12}^	0.02158^{16}^	0.01144^{8}^	0.0159^{15}^	0.01268^{11}^	0.01582^{14}^
	∑*Ranks*	15^{1}^	41^{3.5}^	91^{10}^	20^{2}^	64^{7}^	41^{3.5}^	44^{5}^	45^{6}^	100^{12}^	117^{13}^	94^{11}^	144^{16}^	70^{8}^	135^{15}^	77^{9}^	126^{14}^
300	BIAS($\overset{ˆ}{\beta }$)	0.10176^{3}^	0.10488^{5}^	0.11763^{10}^	0.10113^{2}^	0.12082^{11}^	0.10091^{1}^	0.10226^{4}^	0.10817^{6}^	0.1265^{12}^	0.12722^{13}^	0.11445^{9}^	0.22741^{16}^	0.11142^{8}^	0.13443^{15}^	0.11047^{7}^	0.13361^{14}^
	BIAS($\overset{ˆ}{\gamma }$)	0.05511^{1}^	0.05903^{3}^	0.06833^{11}^	0.05738^{2}^	0.06731^{9}^	0.06413^{7}^	0.06377^{6}^	0.06105^{4}^	0.07141^{12}^	0.07708^{13}^	0.06314^{5}^	0.11403^{16}^	0.06792^{10}^	0.07964^{14}^	0.06438^{8}^	0.08308^{15}^
	MSE($\overset{ˆ}{\beta }$)	0.01638^{2}^	0.01749^{5}^	0.02213^{10}^	0.01632^{1}^	0.02296^{11}^	0.01644^{4}^	0.01642^{3}^	0.01861^{6}^	0.02575^{12}^	0.02605^{13}^	0.02065^{8}^	0.08182^{16}^	0.02072^{9}^	0.02904^{15}^	0.01924^{7}^	0.0285^{14}^
	MSE($\overset{ˆ}{\gamma }$)	0.0048^{1}^	0.00554^{3}^	0.00742^{11}^	0.00508^{2}^	0.00726^{9.5}^	0.0063^{6}^	0.00647^{8}^	0.00591^{4}^	0.0079^{12}^	0.00906^{13}^	0.0062^{5}^	0.02049^{16}^	0.00726^{9.5}^	0.00971^{14}^	0.00638^{7}^	0.0109^{15}^
	MRE($\overset{ˆ}{\beta }$)	0.0407^{3}^	0.04195^{5}^	0.04705^{10}^	0.04045^{2}^	0.04833^{11}^	0.04036^{1}^	0.0409^{4}^	0.04327^{6}^	0.0506^{12}^	0.05089^{13}^	0.04578^{9}^	0.09096^{16}^	0.04457^{8}^	0.05377^{15}^	0.04419^{7}^	0.05344^{14}^
	MRE($\overset{ˆ}{\gamma }$)	0.03674^{1}^	0.03935^{3}^	0.04555^{11}^	0.03825^{2}^	0.04487^{9}^	0.04276^{7}^	0.04251^{6}^	0.0407^{4}^	0.04761^{12}^	0.05139^{13}^	0.0421^{5}^	0.07602^{16}^	0.04528^{10}^	0.05309^{14}^	0.04292^{8}^	0.05539^{15}^
	*D*_*abs*_	0.01431^{1}^	0.01469^{2}^	0.01607^{8}^	0.0147^{3}^	0.01611^{9}^	0.01507^{5}^	0.01526^{6}^	0.01505^{4}^	0.01614^{10}^	0.0194^{13}^	0.01657^{11.5}^	0.02245^{16}^	0.01536^{7}^	0.01998^{15}^	0.01657^{11.5}^	0.01982^{14}^
	*D*_*max*_	0.02312^{1}^	0.02379^{3}^	0.02625^{8}^	0.02374^{2}^	0.02644^{9}^	0.02444^{4}^	0.02474^{6}^	0.02448^{5}^	0.02675^{12}^	0.03111^{13}^	0.0266^{11}^	0.04025^{16}^	0.02534^{7}^	0.03221^{14}^	0.02651^{10}^	0.03223^{15}^
	ASAE	0.00829^{1}^	0.00808^{3}^	0.00839^{8}^	0.00826^{2}^	0.00835^{9}^	0.00833^{4}^	0.008^{6}^	0.00798^{5}^	0.00925^{12}^	0.0115^{13}^	0.01055^{11}^	0.01816^{16}^	0.00896^{7}^	0.01276^{14}^	0.00984^{10}^	0.01289^{15}^
	∑*Ranks*	18^{1}^	32^{3}^	87^{11}^	20^{2}^	85.5^{10}^	41^{5}^	45^{6}^	40^{4}^	104^{12}^	117^{13}^	75.5^{7}^	144^{16}^	77.5^{9}^	130^{14}^	76.5^{8}^	131^{15}^
400	BIAS($\overset{ˆ}{\beta }$)	0.08947^{3}^	0.08951^{4}^	0.10671^{12}^	0.08455^{1}^	0.09476^{7}^	0.08767^{2}^	0.09308^{6}^	0.09292^{5}^	0.10556^{11}^	0.10973^{13}^	0.10269^{10}^	0.19611^{16}^	0.09748^{8}^	0.11285^{14}^	0.09971^{9}^	0.11698^{15}^
	BIAS($\overset{ˆ}{\gamma }$)	0.04975^{1}^	0.05272^{4}^	0.06134^{12}^	0.05199^{3}^	0.05984^{10}^	0.05602^{6}^	0.05382^{5}^	0.05163^{2}^	0.06107^{11}^	0.06145^{13}^	0.05823^{8}^	0.10058^{16}^	0.05846^{9}^	0.07184^{15}^	0.05694^{7}^	0.06989^{14}^
	MSE($\overset{ˆ}{\beta }$)	0.01269^{3}^	0.01287^{4}^	0.01827^{12}^	0.01126^{1}^	0.01448^{7}^	0.01202^{2}^	0.0135^{5}^	0.01386^{6}^	0.01733^{11}^	0.0187^{13}^	0.01562^{9.5}^	0.06103^{16}^	0.01562^{9.5}^	0.02057^{14}^	0.01549^{8}^	0.02157^{15}^
	MSE($\overset{ˆ}{\gamma }$)	0.0041^{1}^	0.00436^{4}^	0.00616^{13}^	0.00419^{2}^	0.00556^{10}^	0.00483^{6}^	0.00449^{5}^	0.00425^{3}^	0.00572^{11}^	0.00605^{12}^	0.00514^{8}^	0.01556^{16}^	0.00553^{9}^	0.00799^{15}^	0.00502^{7}^	0.00758^{14}^
	MRE($\overset{ˆ}{\beta }$)	0.03579^{3}^	0.0358^{4}^	0.04269^{12}^	0.03382^{1}^	0.0379^{7}^	0.03507^{2}^	0.03723^{6}^	0.03717^{5}^	0.04223^{11}^	0.04389^{13}^	0.04107^{10}^	0.07844^{16}^	0.03899^{8}^	0.04514^{14}^	0.03989^{9}^	0.04679^{15}^
	MRE($\overset{ˆ}{\gamma }$)	0.03316^{1}^	0.03515^{4}^	0.0409^{12}^	0.03466^{3}^	0.03989^{10}^	0.03735^{6}^	0.03588^{5}^	0.03442^{2}^	0.04071^{11}^	0.04097^{13}^	0.03882^{8}^	0.06705^{16}^	0.03897^{9}^	0.0479^{15}^	0.03796^{7}^	0.04659^{14}^
	*D*_*abs*_	0.01267^{2}^	0.01242^{1}^	0.01423^{10}^	0.01294^{3}^	0.01333^{7}^	0.01346^{9}^	0.01315^{5}^	0.01313^{4}^	0.01334^{8}^	0.01598^{13}^	0.01467^{12}^	0.01918^{16}^	0.01331^{6}^	0.01722^{15}^	0.0145^{11}^	0.01699^{14}^
	*D*_*max*_	0.02043^{2}^	0.02032^{1}^	0.02342^{11}^	0.0208^{3}^	0.02192^{7.5}^	0.02172^{6}^	0.02141^{5}^	0.02122^{4}^	0.02213^{9}^	0.02564^{13}^	0.02365^{12}^	0.03443^{16}^	0.02192^{7.5}^	0.02783^{15}^	0.0234^{10}^	0.02752^{14}^
	ASAE	0.00696^{2}^	0.00679^{1}^	0.00712^{11}^	0.00698^{3}^	0.00723^{7.5}^	0.00704^{6}^	0.00669^{5}^	0.00683^{4}^	0.00775^{9}^	0.00963^{13}^	0.00905^{12}^	0.01528^{16}^	0.00761^{7.5}^	0.01067^{15}^	0.00836^{10}^	0.01067^{14}^
	∑*Ranks*	20^{1}^	28^{3}^	101^{12}^	22^{2}^	73.5^{7}^	45^{6}^	43^{5}^	34^{4}^	93^{11}^	116^{13}^	89.5^{10}^	144^{16}^	75^{8}^	132^{15}^	79^{9}^	129^{14}^

**Table 2:** Numerical values of simulation measures for *β* = 0.5, *γ* = 0.3.

n	Est.	MLE	ADE	CVME	MPSE	OLSE	PCE	RTADE	WLSE	LTADE	MSADE	MSALDE	ADSOE	KE	MSSD	MSSLD	MSLND
20	BIAS($\overset{ˆ}{\beta }$)	0.12769^{2}^	0.14056^{11}^	0.13645^{7}^	0.13586^{6}^	0.13717^{8}^	0.13364^{3}^	0.14209^{12}^	0.13741^{9}^	0.13819^{10}^	0.13534^{5}^	0.1483^{14}^	0.13459^{4}^	0.11112^{1}^	0.15608^{15}^	0.14686^{13}^	0.15767^{16}^
	BIAS($\overset{ˆ}{\gamma }$)	0.48975^{7}^	0.49162^{8}^	0.58267^{14}^	0.46352^{3}^	0.50729^{9}^	0.46648^{4}^	0.52005^{11}^	0.51298^{10}^	0.58202^{13}^	0.37729^{2}^	0.48171^{6}^	0.69334^{16}^	0.21186^{1}^	0.61655^{15}^	0.47938^{5}^	0.57733^{12}^
	MSE($\overset{ˆ}{\beta }$)	0.02567^{2}^	0.02966^{9}^	0.02932^{7}^	0.02877^{5}^	0.02907^{6}^	0.02809^{3}^	0.03102^{12}^	0.03029^{11}^	0.02947^{8}^	0.03025^{10}^	0.03465^{14}^	0.02854^{4}^	0.02011^{1}^	0.03687^{15}^	0.03374^{13}^	0.03819^{16}^
	MSE($\overset{ˆ}{\gamma }$)	0.42825^{9}^	0.39592^{7}^	0.60185^{15}^	0.3206^{3}^	0.42394^{8}^	0.35227^{4}^	0.49129^{12}^	0.44284^{10}^	0.57565^{13}^	0.30711^{2}^	0.35293^{5}^	0.79529^{16}^	0.123^{1}^	0.58351^{14}^	0.3711^{6}^	0.4784^{11}^
	MRE($\overset{ˆ}{\beta }$)	0.25539^{2}^	0.28112^{11}^	0.2729^{7}^	0.27172^{6}^	0.27434^{8}^	0.26727^{3}^	0.28419^{12}^	0.27482^{9}^	0.27639^{10}^	0.27067^{5}^	0.2966^{14}^	0.26919^{4}^	0.22224^{1}^	0.31216^{15}^	0.29371^{13}^	0.31533^{16}^
	MRE($\overset{ˆ}{\gamma }$)	0.16325^{7}^	0.16387^{8}^	0.19422^{14}^	0.15451^{3}^	0.1691^{9}^	0.15549^{4}^	0.17335^{11}^	0.17099^{10}^	0.19401^{13}^	0.12576^{2}^	0.16057^{6}^	0.23111^{16}^	0.07062^{1}^	0.20552^{15}^	0.15979^{5}^	0.19244^{12}^
	*D*_*abs*_	0.05149^{1}^	0.05754^{6}^	0.0574^{5}^	0.05394^{2}^	0.05896^{10}^	0.05762^{7}^	0.05786^{8}^	0.05703^{4}^	0.05877^{9}^	0.06235^{13}^	0.06213^{12}^	0.06694^{14}^	0.05519^{3}^	0.07083^{16}^	0.05974^{11}^	0.06904^{15}^
	*D*_*max*_	0.08572^{3}^	0.09243^{6}^	0.09682^{10}^	0.08478^{2}^	0.09457^{9}^	0.0907^{4}^	0.09392^{7}^	0.0923^{5}^	0.09862^{12}^	0.09763^{11}^	0.09903^{13}^	0.11679^{16}^	0.0832^{1}^	0.11475^{15}^	0.0943^{8}^	0.11047^{14}^
	ASAE	0.04598^{3}^	0.0415^{6}^	0.04322^{10}^	0.04282^{2}^	0.04131^{9}^	0.04096^{4}^	0.04406^{7}^	0.04042^{5}^	0.04417^{12}^	0.05464^{11}^	0.05232^{13}^	0.05959^{16}^	0.04937^{1}^	0.06037^{15}^	0.04874^{8}^	0.06084^{14}^
	∑*Ranks*	42^{4}^	70^{7.5}^	85^{10}^	35^{3}^	70^{7.5}^	34^{2}^	92^{11}^	69^{6}^	96^{12.5}^	63^{5}^	96^{12.5}^	104^{14}^	21^{1}^	135^{16}^	84^{9}^	128^{15}^
80	BIAS($\overset{ˆ}{\beta }$)	0.06567^{2}^	0.06914^{3}^	0.07651^{11}^	0.06964^{4}^	0.0737^{9}^	0.07198^{7}^	0.07199^{8}^	0.0702^{5}^	0.07395^{10}^	0.07781^{12}^	0.08289^{14}^	0.07142^{6}^	0.059^{1}^	0.08912^{15}^	0.0798^{13}^	0.09231^{16}^
	BIAS($\overset{ˆ}{\gamma }$)	0.22047^{2}^	0.23831^{7}^	0.28381^{13}^	0.23197^{5}^	0.26811^{11}^	0.23187^{4}^	0.2528^{9}^	0.23724^{6}^	0.28087^{12}^	0.23019^{3}^	0.264^{10}^	0.40405^{16}^	0.14916^{1}^	0.30605^{14}^	0.24109^{8}^	0.31657^{15}^
	MSE($\overset{ˆ}{\beta }$)	0.00677^{2}^	0.00771^{3}^	0.00898^{11}^	0.00774^{4}^	0.00838^{8}^	0.00813^{6}^	0.00843^{9}^	0.00782^{5}^	0.00846^{10}^	0.01042^{13}^	0.01103^{14}^	0.00826^{7}^	0.00566^{1}^	0.01262^{15}^	0.00982^{12}^	0.01364^{16}^
	MSE($\overset{ˆ}{\gamma }$)	0.07912^{2}^	0.09049^{6}^	0.12642^{13}^	0.08231^{4}^	0.11252^{11}^	0.08173^{3}^	0.103^{9}^	0.08851^{5}^	0.12357^{12}^	0.10139^{8}^	0.10457^{10}^	0.26727^{16}^	0.05373^{1}^	0.13689^{14}^	0.09063^{7}^	0.15205^{15}^
	MRE($\overset{ˆ}{\beta }$)	0.13133^{2}^	0.13827^{3}^	0.15303^{11}^	0.13929^{4}^	0.14741^{9}^	0.14397^{7}^	0.14399^{8}^	0.1404^{5}^	0.1479^{10}^	0.15561^{12}^	0.16578^{14}^	0.14284^{6}^	0.118^{1}^	0.17823^{15}^	0.15959^{13}^	0.18461^{16}^
	MRE($\overset{ˆ}{\gamma }$)	0.07349^{2}^	0.07944^{7}^	0.0946^{13}^	0.07732^{5}^	0.08937^{11}^	0.07729^{4}^	0.08427^{9}^	0.07908^{6}^	0.09362^{12}^	0.07673^{3}^	0.088^{10}^	0.13468^{16}^	0.04972^{1}^	0.10202^{14}^	0.08036^{8}^	0.10552^{15}^
	*D*_*abs*_	0.02644^{1}^	0.02871^{4}^	0.03107^{9}^	0.02838^{3}^	0.02929^{8}^	0.02908^{6}^	0.02882^{5}^	0.02927^{7}^	0.03161^{10}^	0.03367^{12}^	0.03383^{13}^	0.03864^{15}^	0.02823^{2}^	0.03677^{14}^	0.03235^{11}^	0.03908^{16}^
	*D*_*max*_	0.04329^{1}^	0.04689^{5}^	0.05132^{9}^	0.04568^{3}^	0.04805^{8}^	0.04658^{4}^	0.04704^{6}^	0.04741^{7}^	0.05257^{11}^	0.05395^{12}^	0.0543^{13}^	0.06889^{16}^	0.0444^{2}^	0.05947^{14}^	0.05139^{10}^	0.06349^{15}^
	ASAE	0.01879^{1}^	0.01789^{5}^	0.01826^{9}^	0.01868^{3}^	0.01806^{8}^	0.01823^{4}^	0.0184^{6}^	0.01753^{7}^	0.01883^{11}^	0.02508^{12}^	0.02365^{13}^	0.03029^{16}^	0.02064^{2}^	0.02667^{14}^	0.02183^{10}^	0.02775^{15}^
	∑*Ranks*	22^{2}^	40^{4}^	95^{11}^	39^{3}^	78^{8}^	45^{5}^	69^{7}^	47^{6}^	96^{12}^	88^{9}^	110^{13}^	114^{14}^	20^{1}^	129^{15}^	93^{10}^	139^{16}^
150	BIAS($\overset{ˆ}{\beta }$)	0.04972^{3}^	0.05304^{8}^	0.05124^{6}^	0.0492^{2}^	0.05331^{10}^	0.05104^{5}^	0.05322^{9}^	0.05021^{4}^	0.05274^{7}^	0.05945^{14}^	0.05919^{13}^	0.05487^{11}^	0.04333^{1}^	0.0695^{16}^	0.05659^{12}^	0.06583^{15}^
	BIAS($\overset{ˆ}{\gamma }$)	0.17065^{5}^	0.17183^{6}^	0.19236^{11}^	0.16528^{3}^	0.19476^{12}^	0.16211^{2}^	0.1797^{9}^	0.16868^{4}^	0.20272^{13}^	0.17863^{8}^	0.18703^{10}^	0.3074^{16}^	0.11088^{1}^	0.23762^{15}^	0.17274^{7}^	0.22867^{14}^
	MSE($\overset{ˆ}{\beta }$)	0.00386^{3}^	0.00419^{6}^	0.00429^{7}^	0.00379^{2}^	0.00448^{9}^	0.00402^{4}^	0.00456^{10}^	0.00407^{5}^	0.00444^{8}^	0.00585^{14}^	0.0055^{13}^	0.00459^{11}^	0.00309^{1}^	0.00781^{16}^	0.00501^{12}^	0.0069^{15}^
	MSE($\overset{ˆ}{\gamma }$)	0.0457^{4}^	0.04701^{7}^	0.06241^{12}^	0.04148^{3}^	0.06042^{11}^	0.04072^{2}^	0.05147^{8}^	0.04666^{6}^	0.06619^{13}^	0.05701^{10}^	0.05359^{9}^	0.14601^{16}^	0.02632^{1}^	0.08634^{15}^	0.04637^{5}^	0.07964^{14}^
	MRE($\overset{ˆ}{\beta }$)	0.09944^{3}^	0.10608^{8}^	0.10247^{6}^	0.0984^{2}^	0.10661^{10}^	0.10208^{5}^	0.10644^{9}^	0.10042^{4}^	0.10548^{7}^	0.1189^{14}^	0.11837^{13}^	0.10974^{11}^	0.08665^{1}^	0.139^{16}^	0.11318^{12}^	0.13165^{15}^
	MRE($\overset{ˆ}{\gamma }$)	0.05688^{5}^	0.05728^{6}^	0.06412^{11}^	0.05509^{3}^	0.06492^{12}^	0.05404^{2}^	0.0599^{9}^	0.05623^{4}^	0.06757^{13}^	0.05954^{8}^	0.06234^{10}^	0.10247^{16}^	0.03696^{1}^	0.07921^{15}^	0.05758^{7}^	0.07622^{14}^
	*D*_*abs*_	0.01984^{1}^	0.02174^{8}^	0.0214^{7}^	0.02032^{2}^	0.0222^{9}^	0.02128^{6}^	0.02111^{5}^	0.02083^{4}^	0.02258^{10}^	0.02556^{13}^	0.02406^{12}^	0.03057^{16}^	0.02047^{3}^	0.02767^{15}^	0.0229^{11}^	0.02698^{14}^
	*D*_*max*_	0.03247^{2}^	0.03509^{7}^	0.03551^{8}^	0.03293^{3}^	0.0364^{9}^	0.03405^{5}^	0.03437^{6}^	0.03387^{4}^	0.03766^{11}^	0.04084^{13}^	0.03855^{12}^	0.05426^{16}^	0.03244^{1}^	0.0451^{15}^	0.0367^{10}^	0.04403^{14}^
	ASAE	0.01275^{2}^	0.01217^{7}^	0.0125^{8}^	0.01316^{3}^	0.01237^{9}^	0.0125^{5}^	0.01281^{6}^	0.01223^{4}^	0.01297^{11}^	0.01771^{13}^	0.01629^{12}^	0.02177^{16}^	0.01426^{1}^	0.01912^{15}^	0.01536^{10}^	0.01878^{14}^
	∑*Ranks*	32^{3}^	57^{6}^	73^{8}^	29^{2}^	85^{9}^	35^{4}^	72^{7}^	37^{5}^	90^{11}^	107^{13}^	104^{12}^	129^{14.5}^	20^{1}^	138^{16}^	87^{10}^	129^{14.5}^
200	BIAS($\overset{ˆ}{\beta }$)	0.04364^{3}^	0.04455^{5}^	0.04835^{11}^	0.04433^{4}^	0.04742^{10}^	0.04119^{2}^	0.04692^{9}^	0.04612^{7}^	0.04566^{6}^	0.0541^{14}^	0.051^{13}^	0.04631^{8}^	0.03862^{1}^	0.05815^{15}^	0.04985^{12}^	0.05901^{16}^
	BIAS($\overset{ˆ}{\gamma }$)	0.14504^{3}^	0.14607^{4}^	0.173^{12}^	0.14931^{5}^	0.17025^{11}^	0.13526^{2}^	0.15888^{7}^	0.15685^{6}^	0.175^{13}^	0.163^{9}^	0.16481^{10}^	0.27326^{16}^	0.09694^{1}^	0.1949^{14}^	0.16165^{8}^	0.19984^{15}^
	MSE($\overset{ˆ}{\beta }$)	0.003^{3}^	0.0031^{5}^	0.00366^{11}^	0.00309^{4}^	0.00357^{10}^	0.0027^{2}^	0.00353^{9}^	0.0033^{7.5}^	0.00326^{6}^	0.0048^{14}^	0.00421^{13}^	0.0033^{7.5}^	0.00243^{1}^	0.00539^{15}^	0.00406^{12}^	0.00543^{16}^
	MSE($\overset{ˆ}{\gamma }$)	0.03318^{3}^	0.03343^{4}^	0.04721^{12}^	0.03392^{5}^	0.0456^{10}^	0.02838^{2}^	0.04115^{8}^	0.03897^{6}^	0.04864^{13}^	0.04651^{11}^	0.04214^{9}^	0.11728^{16}^	0.02075^{1}^	0.06092^{14}^	0.04107^{7}^	0.06152^{15}^
	MRE($\overset{ˆ}{\beta }$)	0.08727^{3}^	0.0891^{5}^	0.09669^{11}^	0.08866^{4}^	0.09484^{10}^	0.08237^{2}^	0.09384^{9}^	0.09223^{7}^	0.09131^{6}^	0.10821^{14}^	0.102^{13}^	0.09262^{8}^	0.07725^{1}^	0.1163^{15}^	0.09971^{12}^	0.11802^{16}^
	MRE($\overset{ˆ}{\gamma }$)	0.04835^{3}^	0.04869^{4}^	0.05767^{12}^	0.04977^{5}^	0.05675^{11}^	0.04509^{2}^	0.05296^{7}^	0.05228^{6}^	0.05833^{13}^	0.05433^{9}^	0.05494^{10}^	0.09109^{16}^	0.03231^{1}^	0.06497^{14}^	0.05388^{8}^	0.06661^{15}^
	*D*_*abs*_	0.01729^{1}^	0.01841^{5}^	0.0197^{9}^	0.01753^{3}^	0.01945^{8}^	0.01733^{2}^	0.01844^{6.5}^	0.01844^{6.5}^	0.01977^{10}^	0.02225^{13}^	0.02117^{12}^	0.02614^{16}^	0.01799^{4}^	0.02456^{15}^	0.02004^{11}^	0.02441^{14}^
	*D*_*max*_	0.02814^{2}^	0.02973^{5}^	0.03233^{10}^	0.0284^{4}^	0.03186^{8}^	0.02786^{1}^	0.02995^{6}^	0.02997^{7}^	0.03276^{11}^	0.03558^{13}^	0.03404^{12}^	0.04676^{16}^	0.02836^{3}^	0.03985^{15}^	0.03228^{9}^	0.03962^{14}^
	ASAE	0.0109^{2}^	0.01033^{5}^	0.01067^{10}^	0.01089^{4}^	0.01065^{8}^	0.01073^{1}^	0.01084^{6}^	0.01048^{7}^	0.01128^{11}^	0.01488^{13}^	0.0138^{12}^	0.0189^{16}^	0.01197^{3}^	0.01646^{15}^	0.01302^{9}^	0.01636^{14}^
	∑*Ranks*	29^{3}^	38^{4}^	92^{11}^	41^{5}^	81^{8}^	20^{1}^	67.5^{7}^	55^{6}^	87^{9}^	110^{13}^	104^{12}^	119.5^{14}^	23^{2}^	132^{15}^	90^{10}^	135^{16}^
300	BIAS($\overset{ˆ}{\beta }$)	0.03475^{3}^	0.03579^{4}^	0.03717^{8}^	0.03428^{2}^	0.03699^{6}^	0.03663^{5}^	0.03862^{11}^	0.03714^{7}^	0.03787^{9}^	0.04289^{14}^	0.04243^{13}^	0.03824^{10}^	0.03253^{1}^	0.04844^{15}^	0.03972^{12}^	0.04862^{16}^
	BIAS($\overset{ˆ}{\gamma }$)	0.11636^{4}^	0.12216^{5}^	0.13273^{11}^	0.11277^{2}^	0.13495^{12}^	0.11582^{3}^	0.12896^{8}^	0.12232^{6}^	0.14127^{13}^	0.13026^{9}^	0.13089^{10}^	0.2252^{16}^	0.08645^{1}^	0.15771^{14}^	0.12535^{7}^	0.16117^{15}^
	MSE($\overset{ˆ}{\beta }$)	0.00187^{2}^	0.00202^{4}^	0.00211^{5}^	0.0019^{3}^	0.00214^{7}^	0.00213^{6}^	0.00233^{11}^	0.00216^{8}^	0.00224^{9}^	0.00309^{14}^	0.00281^{13}^	0.0023^{10}^	0.00173^{1}^	0.00376^{15.5}^	0.00251^{12}^	0.00376^{15.5}^
	MSE($\overset{ˆ}{\gamma }$)	0.02095^{4}^	0.02281^{5}^	0.02777^{10}^	0.02005^{2}^	0.02852^{11}^	0.02082^{3}^	0.02559^{8}^	0.02361^{6}^	0.03235^{13}^	0.02947^{12}^	0.02752^{9}^	0.08359^{16}^	0.01672^{1}^	0.04042^{15}^	0.02488^{7}^	0.04028^{14}^
	MRE($\overset{ˆ}{\beta }$)	0.06949^{3}^	0.07159^{4}^	0.07434^{8}^	0.06856^{2}^	0.07398^{6}^	0.07325^{5}^	0.07724^{11}^	0.07427^{7}^	0.07575^{9}^	0.08579^{14}^	0.08486^{13}^	0.07649^{10}^	0.06507^{1}^	0.09687^{15}^	0.07944^{12}^	0.09724^{16}^
	MRE($\overset{ˆ}{\gamma }$)	0.03879^{4}^	0.04072^{5}^	0.04424^{11}^	0.03759^{2}^	0.04498^{12}^	0.03861^{3}^	0.04299^{8}^	0.04077^{6}^	0.04709^{13}^	0.04342^{9}^	0.04363^{10}^	0.07507^{16}^	0.02882^{1}^	0.05257^{14}^	0.04178^{7}^	0.05372^{15}^
	*D*_*abs*_	0.01393^{1}^	0.01461^{3}^	0.01553^{9}^	0.01406^{2}^	0.01537^{8}^	0.01515^{6}^	0.01524^{7}^	0.01512^{5}^	0.01608^{10}^	0.0181^{13}^	0.01737^{12}^	0.02217^{16}^	0.01502^{4}^	0.02025^{15}^	0.01625^{11}^	0.01997^{14}^
	*D*_*max*_	0.02263^{1}^	0.02385^{4}^	0.02547^{9}^	0.02271^{2}^	0.02533^{8}^	0.02426^{5}^	0.02466^{7}^	0.02455^{6}^	0.02656^{11}^	0.02908^{13}^	0.02781^{12}^	0.03953^{16}^	0.0238^{3}^	0.03269^{15}^	0.0261^{10}^	0.03241^{14}^
	ASAE	0.00844^{1}^	0.00822^{4}^	0.00839^{9}^	0.00859^{2}^	0.00846^{8}^	0.00828^{5}^	0.00865^{7}^	0.00821^{6}^	0.00877^{11}^	0.01182^{13}^	0.01092^{12}^	0.01529^{16}^	0.00939^{3}^	0.01312^{15}^	0.01017^{10}^	0.01298^{14}^
	∑*Ranks*	27^{3}^	36^{4}^	75^{7}^	24^{2}^	76^{8}^	39^{5}^	79^{9}^	52^{6}^	96^{11}^	111^{13}^	104^{12}^	126^{14}^	23^{1}^	133.5^{15.5}^	89^{10}^	133.5^{15.5}^
400	BIAS($\overset{ˆ}{\beta }$)	0.03175^{5}^	0.03115^{4}^	0.03284^{11}^	0.03083^{3}^	0.03264^{8}^	0.03021^{2}^	0.03267^{9}^	0.03181^{6}^	0.03262^{7}^	0.03689^{14}^	0.0358^{13}^	0.03281^{10}^	0.02859^{1}^	0.04244^{16}^	0.03532^{12}^	0.04195^{15}^
	BIAS($\overset{ˆ}{\gamma }$)	0.10397^{4}^	0.10478^{5}^	0.12031^{12}^	0.10148^{3}^	0.11386^{11}^	0.09853^{2}^	0.1106^{7}^	0.10886^{6}^	0.12101^{13}^	0.11292^{10}^	0.11078^{8}^	0.1974^{16}^	0.07716^{1}^	0.14231^{15}^	0.11253^{9}^	0.1399^{14}^
	MSE($\overset{ˆ}{\beta }$)	0.00152^{4}^	0.00153^{5}^	0.0017^{11}^	0.0015^{3}^	0.00167^{9}^	0.00144^{2}^	0.00167^{9}^	0.00157^{6}^	0.00163^{7}^	0.00224^{14}^	0.00209^{13}^	0.00167^{9}^	0.00134^{1}^	0.00283^{16}^	0.00198^{12}^	0.00279^{15}^
	MSE($\overset{ˆ}{\gamma }$)	0.01653^{4}^	0.01707^{5}^	0.0225^{12}^	0.01567^{3}^	0.02084^{10}^	0.01491^{2}^	0.0197^{9}^	0.01865^{6}^	0.02333^{13}^	0.02168^{11}^	0.01934^{7}^	0.06293^{16}^	0.01224^{1}^	0.03093^{15}^	0.01964^{8}^	0.02998^{14}^
	MRE($\overset{ˆ}{\beta }$)	0.0635^{5}^	0.0623^{4}^	0.06567^{11}^	0.06167^{3}^	0.06528^{8}^	0.06041^{2}^	0.06533^{9}^	0.06362^{6}^	0.06524^{7}^	0.07379^{14}^	0.07161^{13}^	0.06561^{10}^	0.05718^{1}^	0.08488^{16}^	0.07064^{12}^	0.0839^{15}^
	MRE($\overset{ˆ}{\gamma }$)	0.03466^{4}^	0.03493^{5}^	0.0401^{12}^	0.03383^{3}^	0.03795^{11}^	0.03284^{2}^	0.03687^{7}^	0.03629^{6}^	0.04034^{13}^	0.03764^{10}^	0.03693^{8}^	0.0658^{16}^	0.02572^{1}^	0.04744^{15}^	0.03751^{9}^	0.04663^{14}^
	*D*_*abs*_	0.01266^{4}^	0.01265^{3}^	0.01348^{8}^	0.01261^{2}^	0.01359^{9}^	0.01234^{1}^	0.01309^{6}^	0.01287^{5}^	0.01382^{10}^	0.01563^{13}^	0.01478^{12}^	0.01935^{16}^	0.01322^{7}^	0.01737^{15}^	0.0142^{11}^	0.01693^{14}^
	*D*_*max*_	0.02049^{3}^	0.02056^{4}^	0.02225^{9}^	0.02037^{2}^	0.02222^{8}^	0.01989^{1}^	0.02127^{7}^	0.02092^{5}^	0.02284^{11}^	0.02509^{13}^	0.02368^{12}^	0.03462^{16}^	0.02102^{6}^	0.02817^{15}^	0.02282^{10}^	0.0275^{14}^
	ASAE	0.00731^{3}^	0.00693^{4}^	0.00715^{9}^	0.00733^{2}^	0.00709^{8}^	0.00712^{1}^	0.00731^{7}^	0.00694^{5}^	0.00741^{11}^	0.00999^{13}^	0.00916^{12}^	0.01319^{16}^	0.00793^{6}^	0.01104^{15}^	0.00885^{10}^	0.01094^{14}^
	∑*Ranks*	39^{5}^	36^{4}^	91^{10}^	30^{3}^	77^{8}^	18^{1}^	70^{7}^	48^{6}^	90^{9}^	112^{13}^	98^{12}^	125^{14}^	29^{2}^	138^{16}^	94^{11}^	129^{15}^

**Table 3:** Numerical values of simulation measures for *β* = 2.0, *γ* = 0.7.

n	Est.	MLE	ADE	CVME	MPSE	OLSE	PCE	RTADE	WLSE	LTADE	MSADE	MSALDE	ADSOE	KE	MSSD	MSSLD	MSLND
20	BIAS($\overset{ˆ}{\beta }$)	0.34696^{11}^	0.34593^{8}^	0.34666^{10}^	0.29961^{1}^	0.3476^{12}^	0.34357^{7}^	0.34632^{9}^	0.3332^{4}^	0.3896^{15}^	0.31168^{2}^	0.34183^{6}^	0.46129^{16}^	0.32613^{3}^	0.36264^{14}^	0.34826^{13}^	0.33718^{5}^
	BIAS($\overset{ˆ}{\gamma }$)	0.1149^{4}^	0.11325^{3}^	0.12845^{8}^	0.10692^{1}^	0.13084^{9}^	0.16656^{16}^	0.11853^{5}^	0.11957^{6}^	0.13549^{11}^	0.13299^{10}^	0.12172^{7}^	0.15426^{15}^	0.13617^{12}^	0.15129^{14}^	0.1122^{2}^	0.14371^{13}^
	MSE($\overset{ˆ}{\beta }$)	0.20995^{12}^	0.195^{7}^	0.21336^{13}^	0.15317^{1}^	0.20384^{10}^	0.19001^{6}^	0.20637^{11}^	0.18718^{5}^	0.26101^{15}^	0.17769^{2}^	0.19782^{8}^	0.37802^{16}^	0.18144^{3}^	0.21727^{14}^	0.20246^{9}^	0.18548^{4}^
	MSE($\overset{ˆ}{\gamma }$)	0.02247^{5}^	0.02116^{3}^	0.02932^{10}^	0.01703^{1}^	0.02834^{9}^	0.04314^{16}^	0.02386^{6}^	0.02454^{7}^	0.03087^{12}^	0.028^{8}^	0.02238^{4}^	0.03723^{15}^	0.0322^{13}^	0.0343^{14}^	0.0198^{2}^	0.0308^{11}^
	MRE($\overset{ˆ}{\beta }$)	0.17348^{11}^	0.17297^{8}^	0.17333^{10}^	0.14981^{1}^	0.1738^{12}^	0.17178^{7}^	0.17316^{9}^	0.1666^{4}^	0.1948^{15}^	0.15584^{2}^	0.17091^{6}^	0.23064^{16}^	0.16307^{3}^	0.18132^{14}^	0.17413^{13}^	0.16859^{5}^
	MRE($\overset{ˆ}{\gamma }$)	0.16414^{4}^	0.16178^{3}^	0.1835^{8}^	0.15274^{1}^	0.18691^{9}^	0.23794^{16}^	0.16932^{5}^	0.17082^{6}^	0.19355^{11}^	0.18999^{10}^	0.17388^{7}^	0.22037^{15}^	0.19453^{12}^	0.21612^{14}^	0.16029^{2}^	0.20531^{13}^
	*D*_*abs*_	0.05575^{1}^	0.05975^{7}^	0.05715^{2}^	0.05757^{3}^	0.0626^{9}^	0.07847^{16}^	0.05955^{6}^	0.05929^{5}^	0.06271^{10}^	0.06348^{11}^	0.06517^{12}^	0.06582^{13}^	0.05859^{4}^	0.07277^{15}^	0.06184^{8}^	0.06862^{14}^
	*D*_*max*_	0.09082^{2}^	0.09496^{4}^	0.09423^{3}^	0.09014^{1}^	0.10064^{9}^	0.1267^{16}^	0.09563^{6}^	0.09532^{5}^	0.10279^{11}^	0.10221^{10}^	0.10328^{12}^	0.11237^{14}^	0.09579^{7}^	0.11675^{15}^	0.09786^{8}^	0.11046^{13}^
	ASAE	0.04538^{2}^	0.04322^{4}^	0.04504^{3}^	0.04113^{1}^	0.04665^{9}^	0.03715^{16}^	0.03891^{6}^	0.04243^{5}^	0.05405^{11}^	0.05014^{10}^	0.0485^{12}^	0.09914^{14}^	0.04923^{7}^	0.06738^{15}^	0.04713^{8}^	0.06545^{13}^
	∑*Ranks*	57^{4}^	48^{3}^	70^{9}^	13^{1}^	87^{11}^	101^{13}^	59^{5}^	46^{2}^	113^{14}^	67^{7}^	72^{10}^	136^{16}^	68^{8}^	129^{15}^	66^{6}^	92^{12}^
80	BIAS($\overset{ˆ}{\beta }$)	0.16737^{5}^	0.16529^{4}^	0.17605^{9}^	0.1514^{1}^	0.16224^{2}^	0.18285^{11}^	0.16776^{6}^	0.16878^{7}^	0.18429^{12}^	0.1864^{13}^	0.18176^{10}^	0.27947^{16}^	0.1705^{8}^	0.20069^{15}^	0.16304^{3}^	0.19701^{14}^
	BIAS($\overset{ˆ}{\gamma }$)	0.05053^{1}^	0.05526^{4}^	0.06435^{10}^	0.05512^{2}^	0.06369^{9}^	0.09607^{16}^	0.05653^{5}^	0.05723^{6}^	0.06362^{8}^	0.06863^{12}^	0.06125^{7}^	0.09221^{15}^	0.06697^{11}^	0.0729^{14}^	0.05524^{3}^	0.07253^{13}^
	MSE($\overset{ˆ}{\beta }$)	0.04509^{5}^	0.04407^{3}^	0.05212^{10}^	0.03571^{1}^	0.04411^{4}^	0.05286^{11}^	0.04557^{6}^	0.04685^{7}^	0.05899^{12}^	0.06012^{13}^	0.05161^{9}^	0.136^{16}^	0.04986^{8}^	0.06709^{15}^	0.04236^{2}^	0.06234^{14}^
	MSE($\overset{ˆ}{\gamma }$)	0.00423^{1}^	0.00481^{3}^	0.00682^{10}^	0.0046^{2}^	0.00648^{8}^	0.01459^{16}^	0.00531^{6}^	0.0052^{5}^	0.00654^{9}^	0.00715^{11}^	0.00576^{7}^	0.0137^{15}^	0.00731^{12}^	0.00801^{13}^	0.00494^{4}^	0.00877^{14}^
	MRE($\overset{ˆ}{\beta }$)	0.08369^{5}^	0.08265^{4}^	0.08803^{9}^	0.0757^{1}^	0.08112^{2}^	0.09142^{11}^	0.08388^{6}^	0.08439^{7}^	0.09214^{12}^	0.0932^{13}^	0.09088^{10}^	0.13973^{16}^	0.08525^{8}^	0.10035^{15}^	0.08152^{3}^	0.0985^{14}^
	MRE($\overset{ˆ}{\gamma }$)	0.07218^{1}^	0.07895^{4}^	0.09193^{10}^	0.07874^{2}^	0.09099^{9}^	0.13725^{16}^	0.08076^{5}^	0.08176^{6}^	0.09089^{8}^	0.09804^{12}^	0.08751^{7}^	0.13173^{15}^	0.09568^{11}^	0.10414^{14}^	0.07892^{3}^	0.10361^{13}^
	*D*_*abs*_	0.02844^{1}^	0.02899^{3}^	0.03059^{9.5}^	0.02865^{2}^	0.02941^{5}^	0.04414^{16}^	0.03002^{7}^	0.02909^{4}^	0.03059^{9.5}^	0.03471^{12}^	0.03372^{11}^	0.03857^{15}^	0.03049^{8}^	0.03755^{14}^	0.02995^{6}^	0.03737^{13}^
	*D*_*max*_	0.04566^{1}^	0.04698^{3}^	0.05051^{8}^	0.04606^{2}^	0.04844^{7}^	0.07178^{16}^	0.04831^{6}^	0.0475^{4}^	0.05074^{10}^	0.0565^{12}^	0.05361^{11}^	0.06826^{15}^	0.05068^{9}^	0.06092^{14}^	0.04801^{5}^	0.06054^{13}^
	ASAE	0.01689^{1}^	0.01686^{3}^	0.01875^{8}^	0.01645^{2}^	0.0186^{7}^	0.01712^{16}^	0.01533^{6}^	0.01664^{4}^	0.02101^{10}^	0.02119^{12}^	0.02024^{11}^	0.0428^{15}^	0.02029^{9}^	0.0251^{14}^	0.01873^{5}^	0.02512^{13}^
	∑*Ranks*	25^{2}^	32^{3}^	84.5^{9}^	15^{1}^	53^{7}^	119^{13}^	48^{5}^	49^{6}^	92.5^{11}^	111^{12}^	82^{8}^	139^{16}^	86^{10}^	128^{15}^	37^{4}^	123^{14}^
150	BIAS($\overset{ˆ}{\beta }$)	0.11607^{3}^	0.118^{4}^	0.1307^{9}^	0.113^{1}^	0.1198^{5}^	0.13392^{11}^	0.12373^{6}^	0.11606^{2}^	0.13421^{12}^	0.13123^{10}^	0.14136^{13}^	0.20259^{16}^	0.12901^{8}^	0.15081^{15}^	0.12676^{7}^	0.14838^{14}^
	BIAS($\overset{ˆ}{\gamma }$)	0.03796^{1}^	0.03888^{2.5}^	0.04488^{9}^	0.03888^{2.5}^	0.04491^{10}^	0.07612^{16}^	0.04399^{7}^	0.04096^{5}^	0.04481^{8}^	0.04922^{12}^	0.04363^{6}^	0.06918^{15}^	0.04681^{11}^	0.05733^{14}^	0.03951^{4}^	0.05461^{13}^
	MSE($\overset{ˆ}{\beta }$)	0.02185^{2}^	0.02214^{3}^	0.02663^{8}^	0.02021^{1}^	0.02328^{5}^	0.02939^{10}^	0.02388^{6}^	0.02229^{4}^	0.02964^{11}^	0.03021^{12}^	0.03165^{13}^	0.06724^{16}^	0.02826^{9}^	0.03798^{15}^	0.02549^{7}^	0.0355^{14}^
	MSE($\overset{ˆ}{\gamma }$)	0.00228^{1}^	0.00235^{3}^	0.00332^{10}^	0.00234^{2}^	0.00319^{8}^	0.0091^{16}^	0.0031^{7}^	0.00268^{5}^	0.00323^{9}^	0.00373^{12}^	0.00296^{6}^	0.00771^{15}^	0.00346^{11}^	0.00515^{14}^	0.00254^{4}^	0.00458^{13}^
	MRE($\overset{ˆ}{\beta }$)	0.05803^{2.5}^	0.059^{4}^	0.06535^{9}^	0.0565^{1}^	0.0599^{5}^	0.06696^{11}^	0.06187^{6}^	0.05803^{2.5}^	0.0671^{12}^	0.06562^{10}^	0.07068^{13}^	0.10129^{16}^	0.06451^{8}^	0.0754^{15}^	0.06338^{7}^	0.07419^{14}^
	MRE($\overset{ˆ}{\gamma }$)	0.05422^{1}^	0.05554^{2}^	0.06411^{9}^	0.05555^{3}^	0.06415^{10}^	0.10875^{16}^	0.06284^{7}^	0.05851^{5}^	0.06402^{8}^	0.07031^{12}^	0.06233^{6}^	0.09884^{15}^	0.06687^{11}^	0.0819^{14}^	0.05644^{4}^	0.07802^{13}^
	*D*_*abs*_	0.02034^{1}^	0.02043^{2}^	0.02248^{8}^	0.02082^{4}^	0.02133^{5}^	0.03313^{16}^	0.0223^{7}^	0.02061^{3}^	0.02212^{6}^	0.0246^{11}^	0.02548^{12}^	0.02934^{15}^	0.02252^{9}^	0.02811^{14}^	0.02259^{10}^	0.02784^{13}^
	*D*_*max*_	0.03288^{1}^	0.03326^{2}^	0.03675^{9}^	0.03344^{3}^	0.03514^{5}^	0.05426^{16}^	0.03627^{7}^	0.03362^{4}^	0.03667^{8}^	0.03997^{11}^	0.04057^{12}^	0.05195^{15}^	0.03712^{10}^	0.04578^{14}^	0.03615^{6}^	0.04527^{13}^
	ASAE	0.0108^{1}^	0.0108^{2}^	0.01196^{9}^	0.01059^{3}^	0.01197^{5}^	0.01173^{16}^	0.01023^{7}^	0.01102^{4}^	0.01385^{8}^	0.01383^{11}^	0.01305^{12}^	0.02873^{15}^	0.01285^{10}^	0.01635^{14}^	0.01267^{6}^	0.01657^{13}^
	∑*Ranks*	15.5^{1}^	26.5^{3}^	78^{8}^	19.5^{2}^	61^{7}^	118^{13}^	54^{5}^	35.5^{4}^	87^{9.5}^	102^{12}^	92^{11}^	139^{16}^	87^{9.5}^	129^{15}^	58^{6}^	122^{14}^
200	BIAS($\overset{ˆ}{\beta }$)	0.10199^{3}^	0.10128^{2}^	0.10945^{9}^	0.09776^{1}^	0.10818^{6}^	0.11805^{12}^	0.10364^{5}^	0.10829^{8}^	0.11599^{11}^	0.11921^{13}^	0.11347^{10}^	0.17594^{16}^	0.10307^{4}^	0.12809^{14}^	0.10825^{7}^	0.13252^{15}^
	BIAS($\overset{ˆ}{\gamma }$)	0.0324^{1}^	0.03546^{5}^	0.03958^{10}^	0.03264^{2}^	0.03805^{7}^	0.06307^{16}^	0.03541^{4}^	0.03463^{3}^	0.04122^{12}^	0.0412^{11}^	0.03892^{8}^	0.05902^{15}^	0.03955^{9}^	0.04698^{14}^	0.0371^{6}^	0.04637^{13}^
	MSE($\overset{ˆ}{\beta }$)	0.01584^{2}^	0.01626^{3}^	0.01918^{8}^	0.01506^{1}^	0.01911^{7}^	0.02341^{12}^	0.01673^{4}^	0.01845^{6}^	0.02189^{11}^	0.02408^{13}^	0.0206^{10}^	0.04939^{16}^	0.01771^{5}^	0.02603^{14}^	0.01928^{9}^	0.02746^{15}^
	MSE($\overset{ˆ}{\gamma }$)	0.00169^{2}^	0.00196^{4}^	0.00253^{10}^	0.00164^{1}^	0.0022^{7}^	0.0066^{16}^	0.00202^{5}^	0.00192^{3}^	0.00271^{11}^	0.00272^{12}^	0.00228^{8}^	0.00566^{15}^	0.00244^{9}^	0.00347^{14}^	0.00213^{6}^	0.0033^{13}^
	MRE($\overset{ˆ}{\beta }$)	0.051^{3}^	0.05064^{2}^	0.05473^{9}^	0.04888^{1}^	0.05409^{6}^	0.05902^{12}^	0.05182^{5}^	0.05415^{8}^	0.05799^{11}^	0.0596^{13}^	0.05673^{10}^	0.08797^{16}^	0.05154^{4}^	0.06404^{14}^	0.05412^{7}^	0.06626^{15}^
	MRE($\overset{ˆ}{\gamma }$)	0.04629^{1}^	0.05066^{5}^	0.05655^{10}^	0.04663^{2}^	0.05435^{7}^	0.0901^{16}^	0.05059^{4}^	0.04947^{3}^	0.05889^{12}^	0.05886^{11}^	0.0556^{8}^	0.08432^{15}^	0.05651^{9}^	0.06712^{14}^	0.053^{6}^	0.06624^{13}^
	*D*_*abs*_	0.01792^{2}^	0.01833^{3}^	0.01924^{8}^	0.0178^{1}^	0.019^{7}^	0.02862^{16}^	0.01861^{4}^	0.01883^{6}^	0.01957^{9}^	0.02177^{12}^	0.0207^{11}^	0.02569^{15}^	0.01869^{5}^	0.02351^{13}^	0.02006^{10}^	0.02405^{14}^
	*D*_*max*_	0.02891^{2}^	0.02965^{3}^	0.03157^{8}^	0.02849^{1}^	0.03121^{7}^	0.04649^{16}^	0.03012^{4}^	0.03045^{5}^	0.03262^{10}^	0.03532^{12}^	0.03346^{11}^	0.04508^{15}^	0.03089^{6}^	0.03845^{13}^	0.03229^{9}^	0.03906^{14}^
	ASAE	0.00894^{2}^	0.00899^{3}^	0.01009^{8}^	0.00882^{1}^	0.0099^{7}^	0.0098^{16}^	0.00863^{4}^	0.00914^{5}^	0.01162^{10}^	0.01158^{12}^	0.0108^{11}^	0.02325^{15}^	0.01039^{6}^	0.01317^{13}^	0.00999^{9}^	0.01314^{14}^
	∑*Ranks*	19^{2}^	31^{3}^	81^{9}^	12^{1}^	61^{6.5}^	122^{13}^	36^{4}^	47^{5}^	100^{11}^	109^{12}^	87^{10}^	139^{16}^	61^{6.5}^	125^{14}^	68^{8}^	126^{15}^
300	BIAS($\overset{ˆ}{\beta }$)	0.0803^{2}^	0.08503^{4}^	0.09013^{8}^	0.0792^{1}^	0.08648^{6}^	0.09777^{11}^	0.0862^{5}^	0.08204^{3}^	0.09848^{12}^	0.09892^{13}^	0.0949^{10}^	0.15147^{16}^	0.08943^{7}^	0.10931^{15}^	0.09062^{9}^	0.10911^{14}^
	BIAS($\overset{ˆ}{\gamma }$)	0.02671^{1}^	0.02704^{2}^	0.0316^{7}^	0.02725^{3}^	0.03213^{8}^	0.05334^{16}^	0.02836^{4}^	0.02848^{5}^	0.03295^{11}^	0.03353^{12}^	0.03226^{9}^	0.05227^{15}^	0.03253^{10}^	0.03761^{14}^	0.02947^{6}^	0.03677^{13}^
	MSE($\overset{ˆ}{\beta }$)	0.01047^{2}^	0.01142^{4}^	0.01315^{9}^	0.00999^{1}^	0.01179^{6}^	0.01567^{12}^	0.01164^{5}^	0.0106^{3}^	0.0153^{11}^	0.01582^{13}^	0.01448^{10}^	0.03771^{16}^	0.01298^{7}^	0.01917^{15}^	0.01304^{8}^	0.01871^{14}^
	MSE($\overset{ˆ}{\gamma }$)	0.0011^{1}^	0.0012^{3}^	0.0016^{8}^	0.00112^{2}^	0.00164^{9}^	0.00452^{16}^	0.00133^{5}^	0.00131^{4}^	0.00174^{12}^	0.00173^{11}^	0.00158^{7}^	0.00444^{15}^	0.00166^{10}^	0.00223^{14}^	0.00137^{6}^	0.00211^{13}^
	MRE($\overset{ˆ}{\beta }$)	0.04015^{2}^	0.04251^{4}^	0.04506^{8}^	0.0396^{1}^	0.04324^{6}^	0.04889^{11}^	0.0431^{5}^	0.04102^{3}^	0.04924^{12}^	0.04946^{13}^	0.04745^{10}^	0.07574^{16}^	0.04472^{7}^	0.05466^{15}^	0.04531^{9}^	0.05456^{14}^
	MRE($\overset{ˆ}{\gamma }$)	0.03816^{1}^	0.03862^{2}^	0.04514^{7}^	0.03893^{3}^	0.04589^{8}^	0.0762^{16}^	0.04051^{4}^	0.04069^{5}^	0.04708^{11}^	0.04791^{12}^	0.04609^{9}^	0.07467^{15}^	0.04647^{10}^	0.05372^{14}^	0.04209^{6}^	0.05253^{13}^
	*D*_*abs*_	0.01428^{1}^	0.01465^{3}^	0.01573^{7}^	0.01444^{2}^	0.01531^{5}^	0.02365^{16}^	0.01546^{6}^	0.0147^{4}^	0.01616^{10}^	0.01796^{12}^	0.01743^{11}^	0.02194^{15}^	0.01583^{8}^	0.02006^{14}^	0.01605^{9}^	0.01955^{13}^
	*D*_*max*_	0.02316^{1}^	0.02377^{3}^	0.02588^{7}^	0.02326^{2}^	0.02522^{6}^	0.03846^{15}^	0.02488^{5}^	0.02395^{4}^	0.02685^{10}^	0.02902^{12}^	0.02799^{11}^	0.03899^{16}^	0.02614^{9}^	0.03238^{14}^	0.02597^{8}^	0.03179^{13}^
	ASAE	0.00673^{1}^	0.00685^{3}^	0.0076^{7}^	0.00661^{2}^	0.00783^{6}^	0.00764^{15}^	0.00638^{5}^	0.0069^{4}^	0.00861^{10}^	0.00894^{12}^	0.00813^{11}^	0.01896^{16}^	0.00822^{9}^	0.00996^{14}^	0.00793^{8}^	0.01024^{13}^
	∑*Ranks*	14^{1}^	29^{3}^	67^{7}^	17^{2}^	62^{6}^	120^{13}^	40^{5}^	36^{4}^	101^{11}^	111^{12}^	87^{10}^	140^{16}^	79^{9}^	129^{15}^	70^{8}^	122^{14}^
400	BIAS($\overset{ˆ}{\beta }$)	0.07039^{2}^	0.07377^{5}^	0.07437^{6}^	0.06829^{1}^	0.07369^{4}^	0.08684^{12}^	0.0707^{3}^	0.07737^{8}^	0.08011^{9}^	0.08495^{11}^	0.0871^{13}^	0.13808^{16}^	0.0757^{7}^	0.09622^{14}^	0.08018^{10}^	0.09962^{15}^
	BIAS($\overset{ˆ}{\gamma }$)	0.02301^{1}^	0.02466^{4}^	0.02789^{10}^	0.02395^{3}^	0.02699^{8}^	0.04771^{16}^	0.02593^{6}^	0.02376^{2}^	0.02825^{11}^	0.02984^{12}^	0.02681^{7}^	0.04579^{15}^	0.02732^{9}^	0.03184^{13}^	0.02527^{5}^	0.03208^{14}^
	MSE($\overset{ˆ}{\beta }$)	0.008^{3}^	0.00852^{4}^	0.00916^{6}^	0.00714^{1}^	0.00885^{5}^	0.01195^{12}^	0.00798^{2}^	0.00937^{7.5}^	0.01005^{9}^	0.01197^{13}^	0.01169^{11}^	0.0309^{16}^	0.00937^{7.5}^	0.01502^{14}^	0.01046^{10}^	0.01546^{15}^
	MSE($\overset{ˆ}{\gamma }$)	0.00084^{1}^	0.00096^{4}^	0.00119^{9}^	0.00088^{2}^	0.00112^{8}^	0.00357^{16}^	0.00106^{6}^	0.00089^{3}^	0.00123^{10}^	0.00142^{12}^	0.00111^{7}^	0.00346^{15}^	0.00124^{11}^	0.00163^{14}^	0.001^{5}^	0.00158^{13}^
	MRE($\overset{ˆ}{\beta }$)	0.03519^{2}^	0.03689^{5}^	0.03719^{6}^	0.03414^{1}^	0.03684^{4}^	0.04342^{12}^	0.03535^{3}^	0.03868^{8}^	0.04005^{9}^	0.04248^{11}^	0.04355^{13}^	0.06904^{16}^	0.03785^{7}^	0.04811^{14}^	0.04009^{10}^	0.04981^{15}^
	MRE($\overset{ˆ}{\gamma }$)	0.03287^{1}^	0.03522^{4}^	0.03985^{10}^	0.03422^{3}^	0.03856^{8}^	0.06816^{16}^	0.03704^{6}^	0.03395^{2}^	0.04036^{11}^	0.04263^{12}^	0.0383^{7}^	0.06542^{15}^	0.03903^{9}^	0.04549^{13}^	0.0361^{5}^	0.04583^{14}^
	*D*_*abs*_	0.01261^{1}^	0.01307^{3}^	0.01338^{7}^	0.01263^{2}^	0.01312^{4}^	0.02088^{16}^	0.01314^{5}^	0.01328^{6}^	0.01365^{9}^	0.01574^{12}^	0.01533^{11}^	0.01935^{15}^	0.01361^{8}^	0.01725^{13}^	0.01424^{10}^	0.01766^{14}^
	*D*_*max*_	0.02029^{1}^	0.02126^{3}^	0.02199^{7}^	0.02035^{2}^	0.02158^{6}^	0.0341^{15}^	0.02131^{4}^	0.02145^{5}^	0.02256^{9}^	0.02545^{12}^	0.02448^{11}^	0.03481^{16}^	0.02225^{8}^	0.0279^{13}^	0.02289^{10}^	0.02853^{14}^
	ASAE	0.00562^{1}^	0.00593^{3}^	0.00623^{7}^	0.00553^{2}^	0.00628^{6}^	0.00649^{15}^	0.00524^{4}^	0.00577^{5}^	0.00725^{9}^	0.00723^{12}^	0.00684^{11}^	0.01617^{16}^	0.00672^{8}^	0.00854^{13}^	0.00644^{10}^	0.00854^{14}^
	∑*Ranks*	15^{1}^	37^{4}^	67^{7}^	17^{2}^	54^{6}^	124^{14}^	36^{3}^	45.5^{5}^	90^{10}^	107^{12}^	91^{11}^	140^{16}^	76.5^{9}^	122^{13}^	73^{8}^	129^{15}^

**Table 4:** Numerical values of simulation measures for *β* = 0.9, *γ* = 0.2.

n	Est.	MLE	ADE	CVME	MPSE	OLSE	PCE	RTADE	WLSE	LTADE	MSADE	MSALDE	ADSOE	KE	MSSD	MSSLD	MSLND
20	BIAS($\overset{ˆ}{\beta }$)	0.18603^{4}^	0.18844^{7}^	0.1992^{8}^	0.18425^{2}^	0.19928^{9}^	0.34085^{16}^	0.20127^{11}^	0.188^{6}^	0.20529^{13}^	0.18433^{3}^	0.20252^{12}^	0.18747^{5}^	0.17271^{1}^	0.21371^{15}^	0.20068^{10}^	0.2115^{14}^
	BIAS($\overset{ˆ}{\gamma }$)	0.03129^{1}^	0.03205^{4}^	0.03778^{11}^	0.03187^{2}^	0.03715^{10}^	0.06533^{16}^	0.03414^{6}^	0.03425^{7}^	0.03824^{12}^	0.03626^{9}^	0.0335^{5}^	0.04632^{15}^	0.03621^{8}^	0.04493^{14}^	0.0319^{3}^	0.04147^{13}^
	MSE($\overset{ˆ}{\beta }$)	0.05618^{4}^	0.0568^{5}^	0.06223^{9}^	0.05291^{2}^	0.06324^{10}^	0.16318^{16}^	0.0612^{8}^	0.05583^{3}^	0.06904^{13}^	0.06079^{7}^	0.06709^{12}^	0.05894^{6}^	0.05203^{1}^	0.07082^{15}^	0.06481^{11}^	0.07024^{14}^
	MSE($\overset{ˆ}{\gamma }$)	0.00178^{4.5}^	0.00167^{3}^	0.00244^{11}^	0.00152^{1}^	0.00231^{9}^	0.00604^{16}^	0.002^{7}^	0.00191^{6}^	0.00261^{12.5}^	0.00208^{8}^	0.00178^{4.5}^	0.00349^{15}^	0.00236^{10}^	0.00305^{14}^	0.0016^{2}^	0.00261^{12.5}^
	MRE($\overset{ˆ}{\beta }$)	0.2067^{4}^	0.20938^{7}^	0.22133^{8}^	0.20472^{2}^	0.22142^{9}^	0.37872^{16}^	0.22364^{11}^	0.20889^{6}^	0.2281^{13}^	0.20481^{3}^	0.22503^{12}^	0.2083^{5}^	0.1919^{1}^	0.23745^{15}^	0.22298^{10}^	0.23499^{14}^
	MRE($\overset{ˆ}{\gamma }$)	0.15643^{1}^	0.16027^{4}^	0.18889^{11}^	0.15936^{2}^	0.18577^{10}^	0.32667^{16}^	0.17072^{6}^	0.17126^{7}^	0.19121^{12}^	0.18131^{9}^	0.16752^{5}^	0.23162^{15}^	0.18107^{8}^	0.22466^{14}^	0.15948^{3}^	0.20736^{13}^
	*D*_*abs*_	0.0551^{1}^	0.05674^{3}^	0.05928^{7}^	0.05891^{5}^	0.06245^{11}^	0.10012^{16}^	0.05894^{6}^	0.05784^{4}^	0.06243^{10}^	0.06009^{8}^	0.06358^{12}^	0.06642^{13}^	0.05592^{2}^	0.0718^{15}^	0.06101^{9}^	0.07158^{14}^
	*D*_*max*_	0.09002^{1}^	0.09121^{2}^	0.09858^{8}^	0.09237^{3}^	0.10055^{11}^	0.16456^{16}^	0.09512^{6}^	0.09304^{5}^	0.10382^{12}^	0.09891^{9}^	0.10007^{10}^	0.11494^{14}^	0.09293^{4}^	0.11642^{15}^	0.09684^{7}^	0.1142^{13}^
	ASAE	0.04952^{1}^	0.07421^{2}^	0.07596^{8}^	0.06105^{3}^	0.10523^{11}^	0.01695^{16}^	0.04922^{6}^	0.07168^{5}^	0.1221^{12}^	0.05428^{9}^	0.07179^{10}^	3.21794^{14}^	5.28781^{4}^	0.13013^{15}^	0.07887^{7}^	0.14014^{13}^
	∑*Ranks*	23.5^{1}^	43^{3}^	82^{10}^	24^{2}^	90^{11}^	129^{15}^	63^{7}^	50^{4}^	109.5^{13}^	60^{6}^	79.5^{9}^	103^{12}^	51^{5}^	130^{16}^	65^{8}^	121.5^{14}^
80	BIAS($\overset{ˆ}{\beta }$)	0.09487^{5}^	0.09481^{4}^	0.09526^{6}^	0.09876^{9}^	0.09816^{8}^	0.33482^{16}^	0.1015^{10}^	0.0945^{3}^	0.09283^{2}^	0.10287^{11}^	0.10949^{13}^	0.09784^{7}^	0.09112^{1}^	0.12072^{14}^	0.10584^{12}^	0.12505^{15}^
	BIAS($\overset{ˆ}{\gamma }$)	0.01536^{1}^	0.01561^{2}^	0.01795^{9}^	0.01568^{3}^	0.01729^{8}^	0.05023^{16}^	0.01646^{5}^	0.01649^{6}^	0.0181^{11}^	0.01826^{12}^	0.01652^{7}^	0.02711^{15}^	0.01799^{10}^	0.02188^{14}^	0.01619^{4}^	0.02167^{13}^
	MSE($\overset{ˆ}{\beta }$)	0.0143^{5}^	0.01419^{4}^	0.0144^{6}^	0.01529^{7}^	0.01547^{8}^	0.16199^{16}^	0.01637^{10}^	0.01387^{2}^	0.01405^{3}^	0.01864^{12}^	0.01941^{13}^	0.01556^{9}^	0.01375^{1}^	0.024^{14}^	0.01825^{11}^	0.02462^{15}^
	MSE($\overset{ˆ}{\gamma }$)	0.00038^{1.5}^	0.00039^{3}^	5*e* − 04^{9}^	0.00038^{1.5}^	0.00048^{8}^	0.00372^{16}^	0.00044^{6.5}^	0.00044^{6.5}^	0.00053^{10.5}^	0.00054^{12}^	0.00043^{5}^	0.00115^{15}^	0.00053^{10.5}^	0.00073^{14}^	0.00042^{4}^	0.00071^{13}^
	MRE($\overset{ˆ}{\beta }$)	0.10541^{5}^	0.10534^{4}^	0.10585^{6}^	0.10973^{9}^	0.10907^{8}^	0.37202^{16}^	0.11278^{10}^	0.105^{3}^	0.10315^{2}^	0.1143^{11}^	0.12166^{13}^	0.10871^{7}^	0.10124^{1}^	0.13413^{14}^	0.11759^{12}^	0.13895^{15}^
	MRE($\overset{ˆ}{\gamma }$)	0.07678^{1}^	0.07806^{2}^	0.08973^{9}^	0.07841^{3}^	0.08644^{8}^	0.25114^{16}^	0.08231^{5}^	0.08245^{6}^	0.0905^{11}^	0.0913^{12}^	0.08261^{7}^	0.13557^{15}^	0.08997^{10}^	0.10942^{14}^	0.08095^{4}^	0.10834^{13}^
	*D*_*abs*_	0.02852^{1}^	0.02857^{2}^	0.02951^{5}^	0.02993^{7}^	0.03037^{9}^	0.08851^{16}^	0.02975^{6}^	0.02865^{3}^	0.03^{8}^	0.03246^{11}^	0.03352^{12}^	0.03912^{15}^	0.02946^{4}^	0.0387^{14}^	0.03132^{10}^	0.03857^{13}^
	*D*_*max*_	0.04628^{2}^	0.04617^{1}^	0.04908^{7}^	0.04792^{4}^	0.04969^{8}^	0.14246^{16}^	0.04824^{5}^	0.04663^{3}^	0.05021^{10}^	0.0528^{11}^	0.05331^{12}^	0.06904^{15}^	0.04898^{6}^	0.0627^{13}^	0.05017^{9}^	0.06283^{14}^
	ASAE	0.01483^{2}^	0.01832^{1}^	0.02198^{7}^	0.01628^{4}^	0.02436^{8}^	0.00865^{16}^	0.01632^{5}^	0.01852^{3}^	0.02426^{10}^	0.01651^{11}^	0.01687^{12}^	0.50523^{15}^	0.02524^{6}^	0.02616^{13}^	0.01914^{9}^	0.02661^{14}^
	∑*Ranks*	23.5^{1}^	29^{2}^	74^{8}^	46.5^{4}^	77^{10}^	128^{16}^	61.5^{6}^	40.5^{3}^	67.5^{7}^	96^{12}^	88^{11}^	113^{13}^	55.5^{5}^	124^{14}^	75^{9}^	125^{15}^
150	BIAS($\overset{ˆ}{\beta }$)	0.06953^{5}^	0.06841^{3}^	0.07091^{8}^	0.06748^{1}^	0.0689^{4}^	0.31854^{16}^	0.07267^{10}^	0.07148^{9}^	0.07042^{7}^	0.07524^{11}^	0.08208^{13}^	0.07005^{6}^	0.06816^{2}^	0.09105^{15}^	0.07736^{12}^	0.08879^{14}^
	BIAS($\overset{ˆ}{\gamma }$)	0.01091^{2}^	0.01126^{3}^	0.01303^{10}^	0.01084^{1}^	0.01218^{7}^	0.0444^{16}^	0.01164^{4}^	0.01217^{6}^	0.01437^{12}^	0.0134^{11}^	0.01251^{8}^	0.02016^{15}^	0.01291^{9}^	0.01555^{14}^	0.01214^{5}^	0.0154^{13}^
	MSE($\overset{ˆ}{\beta }$)	0.00776^{5}^	0.00717^{1}^	0.00784^{7}^	0.00733^{2}^	0.00763^{3}^	0.15024^{16}^	0.00818^{10}^	0.00814^{9}^	0.00793^{8}^	0.01024^{12}^	0.01061^{13}^	0.00772^{4}^	0.00783^{6}^	0.01321^{15}^	0.00947^{11}^	0.01224^{14}^
	MSE($\overset{ˆ}{\gamma }$)	2*e* − 04^{2.5}^	2*e* − 04^{2.5}^	0.00028^{10.5}^	0.00019^{1}^	0.00024^{7}^	0.00291^{16}^	0.00022^{4}^	0.00024^{7}^	0.00032^{12}^	0.00028^{10.5}^	0.00024^{7}^	0.00067^{15}^	0.00027^{9}^	0.00039^{14}^	0.00023^{5}^	0.00036^{13}^
	MRE($\overset{ˆ}{\beta }$)	0.07725^{5}^	0.07601^{3}^	0.07878^{8}^	0.07498^{1}^	0.07656^{4}^	0.35393^{16}^	0.08075^{10}^	0.07942^{9}^	0.07824^{7}^	0.0836^{11}^	0.09121^{13}^	0.07783^{6}^	0.07573^{2}^	0.10116^{15}^	0.08595^{12}^	0.09865^{14}^
	MRE($\overset{ˆ}{\gamma }$)	0.05454^{2}^	0.0563^{3}^	0.06513^{10}^	0.05421^{1}^	0.0609^{7}^	0.22198^{16}^	0.0582^{4}^	0.06083^{6}^	0.07186^{12}^	0.06702^{11}^	0.06257^{8}^	0.10082^{15}^	0.06453^{9}^	0.07775^{14}^	0.06069^{5}^	0.07702^{13}^
	*D*_*abs*_	0.02048^{1}^	0.0206^{2}^	0.0218^{8}^	0.02073^{3}^	0.02153^{7}^	0.08238^{16}^	0.02137^{4}^	0.02147^{6}^	0.02274^{9}^	0.02352^{11}^	0.02465^{12}^	0.02964^{15}^	0.02144^{5}^	0.02817^{14}^	0.02332^{10}^	0.02766^{13}^
	*D*_*max*_	0.03298^{1}^	0.03335^{3}^	0.03606^{8}^	0.03331^{2}^	0.03523^{6}^	0.13026^{16}^	0.03446^{4}^	0.03487^{5}^	0.03799^{10}^	0.03858^{11}^	0.03948^{12}^	0.05299^{15}^	0.03548^{7}^	0.04572^{14}^	0.03751^{9}^	0.04511^{13}^
	ASAE	0.0087^{1}^	0.01^{3}^	0.01232^{8}^	0.00884^{2}^	0.01226^{6}^	0.00587^{16}^	0.00966^{4}^	0.01102^{5}^	0.01491^{10}^	0.01002^{11}^	0.00974^{12}^	0.08638^{15}^	0.01374^{7}^	0.01357^{14}^	0.01034^{9}^	0.01356^{13}^
	∑*Ranks*	38.5^{2}^	39.5^{3}^	78.5^{9}^	15^{1}^	53^{5}^	127^{16}^	52^{4}^	64^{7}^	90^{11}^	93.5^{12}^	89^{10}^	105^{13}^	61^{6}^	126^{15}^	75^{8}^	117^{14}^
200	BIAS($\overset{ˆ}{\beta }$)	0.06035^{3}^	0.05938^{2}^	0.0638^{10}^	0.0607^{5}^	0.06325^{9}^	0.31347^{16}^	0.06233^{8}^	0.06084^{6}^	0.06037^{4}^	0.06789^{11}^	0.06906^{12}^	0.06178^{7}^	0.05491^{1}^	0.07793^{14}^	0.06961^{13}^	0.08382^{15}^
	BIAS($\overset{ˆ}{\gamma }$)	0.00943^{2}^	0.00957^{3}^	0.01113^{9}^	0.00939^{1}^	0.01172^{11}^	0.04283^{16}^	0.01058^{6}^	0.00996^{4}^	0.01178^{12}^	0.01158^{10}^	0.01103^{7}^	0.0174^{15}^	0.0111^{8}^	0.0136^{14}^	0.01038^{5}^	0.01342^{13}^
	MSE($\overset{ˆ}{\beta }$)	0.0059^{5}^	0.00556^{2}^	0.00652^{10}^	0.00564^{4}^	0.00619^{9}^	0.14729^{16}^	0.00614^{8}^	0.00593^{6}^	0.00563^{3}^	0.00772^{12}^	0.00775^{13}^	0.00599^{7}^	0.00497^{1}^	0.0094^{14}^	0.00767^{11}^	0.01093^{15}^
	MSE($\overset{ˆ}{\gamma }$)	0.00014^{1.5}^	0.00015^{3}^	2*e* − 04^{9}^	0.00014^{1.5}^	0.00021^{10.5}^	0.00269^{16}^	0.00018^{6}^	0.00016^{4}^	0.00022^{12}^	0.00021^{10.5}^	0.00019^{7.5}^	0.00049^{15}^	0.00019^{7.5}^	3*e* − 04^{14}^	0.00017^{5}^	0.00028^{13}^
	MRE($\overset{ˆ}{\beta }$)	0.06705^{3}^	0.06598^{2}^	0.07089^{10}^	0.06745^{5}^	0.07028^{9}^	0.3483^{16}^	0.06925^{8}^	0.0676^{6}^	0.06708^{4}^	0.07543^{11}^	0.07674^{12}^	0.06864^{7}^	0.06101^{1}^	0.08658^{14}^	0.07734^{13}^	0.09313^{15}^
	MRE($\overset{ˆ}{\gamma }$)	0.04717^{2}^	0.04784^{3}^	0.05566^{9}^	0.04696^{1}^	0.05859^{11}^	0.21414^{16}^	0.05292^{6}^	0.04978^{4}^	0.05888^{12}^	0.05792^{10}^	0.05513^{7}^	0.087^{15}^	0.05549^{8}^	0.06802^{14}^	0.05192^{5}^	0.06712^{13}^
	*D*_*abs*_	0.01789^{2}^	0.01769^{1}^	0.01955^{9}^	0.0182^{4}^	0.01939^{8}^	0.08075^{16}^	0.01853^{6}^	0.01835^{5}^	0.01937^{7}^	0.02087^{12}^	0.02047^{10}^	0.02546^{15}^	0.01799^{3}^	0.02401^{13}^	0.02055^{11}^	0.02544^{14}^
	*D*_*max*_	0.02887^{2}^	0.02858^{1}^	0.03204^{8}^	0.02922^{3}^	0.03203^{7}^	0.12699^{16}^	0.03012^{5}^	0.02982^{4}^	0.03218^{9}^	0.03405^{12}^	0.0329^{10}^	0.04486^{15}^	0.03031^{6}^	0.0391^{13}^	0.03294^{11}^	0.04091^{14}^
	ASAE	0.00652^{2}^	0.00823^{1}^	0.00947^{8}^	0.00692^{3}^	0.00979^{7}^	0.00495^{16}^	0.0077^{5}^	0.00815^{4}^	0.01108^{9}^	0.00728^{12}^	0.0076^{10}^	0.03941^{15}^	0.00995^{6}^	0.00981^{13}^	0.00785^{11}^	0.0098^{14}^
	∑*Ranks*	22.5^{1}^	26^{2}^	90^{11}^	27.5^{3}^	84.5^{10}^	127^{16}^	59^{6}^	47^{4}^	77^{7}^	91.5^{12}^	83.5^{9}^	110^{13}^	49.5^{5}^	125^{15}^	81^{8}^	123^{14}^
300	BIAS($\overset{ˆ}{\beta }$)	0.04939^{5}^	0.05034^{9}^	0.04736^{1}^	0.05021^{8}^	0.04898^{4}^	0.29941^{16}^	0.05205^{10}^	0.0496^{6}^	0.04814^{2}^	0.05813^{13}^	0.05461^{11}^	0.04993^{7}^	0.04874^{3}^	0.06494^{14}^	0.05704^{12}^	0.06724^{15}^
	BIAS($\overset{ˆ}{\gamma }$)	0.00744^{1}^	0.00797^{3}^	0.00884^{8}^	0.00781^{2}^	0.00892^{9}^	0.03801^{16}^	0.0083^{5}^	0.00815^{4}^	0.00909^{10}^	0.00938^{12}^	0.00862^{7}^	0.0146^{15}^	0.00934^{11}^	0.01087^{13}^	0.0086^{6}^	0.01093^{14}^
	MSE($\overset{ˆ}{\beta }$)	0.00388^{6}^	0.00401^{9}^	0.00358^{1}^	0.00385^{5}^	0.00377^{3}^	0.13539^{16}^	0.00416^{10}^	0.0038^{4}^	0.00366^{2}^	0.00581^{13}^	0.00486^{11}^	0.00393^{7}^	0.00397^{8}^	0.00677^{14}^	0.0051^{12}^	0.00718^{15}^
	MSE($\overset{ˆ}{\gamma }$)	9*e* − 05^{1.5}^	1*e* − 04^{3}^	0.00012^{7.5}^	9*e* − 05^{1.5}^	0.00012^{7.5}^	0.00213^{16}^	0.00011^{4.5}^	0.00011^{4.5}^	0.00013^{10}^	0.00014^{11.5}^	0.00012^{7.5}^	0.00036^{15}^	0.00014^{11.5}^	0.00018^{13.5}^	0.00012^{7.5}^	0.00018^{13.5}^
	MRE($\overset{ˆ}{\beta }$)	0.05487^{5}^	0.05593^{9}^	0.05262^{1}^	0.05578^{8}^	0.05442^{4}^	0.33268^{16}^	0.05784^{10}^	0.05511^{6}^	0.05349^{2}^	0.06459^{13}^	0.06067^{11}^	0.05547^{7}^	0.05415^{3}^	0.07216^{14}^	0.06338^{12}^	0.07471^{15}^
	MRE($\overset{ˆ}{\gamma }$)	0.03718^{1}^	0.03987^{3}^	0.04419^{8}^	0.03906^{2}^	0.04461^{9}^	0.19007^{16}^	0.04148^{5}^	0.04074^{4}^	0.04547^{10}^	0.04688^{12}^	0.04311^{7}^	0.07298^{15}^	0.0467^{11}^	0.05433^{13}^	0.04299^{6}^	0.05465^{14}^
	*D*_*abs*_	0.0145^{1}^	0.01513^{5}^	0.0147^{2}^	0.01503^{4}^	0.01526^{7}^	0.07581^{16}^	0.01529^{8}^	0.0148^{3}^	0.01524^{6}^	0.01773^{12}^	0.01651^{10}^	0.02148^{15}^	0.01549^{9}^	0.01966^{13}^	0.01689^{11}^	0.02032^{14}^
	*D*_*max*_	0.02334^{1}^	0.02446^{5}^	0.02419^{4}^	0.02418^{3}^	0.02505^{7}^	0.11848^{16}^	0.02469^{6}^	0.02402^{2}^	0.02538^{8}^	0.02873^{12}^	0.02667^{10}^	0.0384^{15}^	0.02564^{9}^	0.03199^{13}^	0.02708^{11}^	0.03292^{14}^
	ASAE	0.0045^{1}^	0.0057^{5}^	0.00629^{4}^	0.00467^{3}^	0.00634^{7}^	0.00377^{16}^	0.00518^{6}^	0.00545^{2}^	0.00704^{8}^	0.00517^{12}^	0.005^{10}^	0.03112^{15}^	0.00687^{9}^	0.00625^{13}^	0.00508^{11}^	0.00663^{14}^
	∑*Ranks*	37.5^{1}^	66^{8}^	40.5^{3}^	50.5^{4}^	59.5^{5}^	126^{16}^	62.5^{7}^	38.5^{2}^	62^{6}^	101.5^{12}^	75.5^{9}^	109^{13}^	76.5^{10}^	114.5^{14}^	79.5^{11}^	124.5^{15}^
400	BIAS($\overset{ˆ}{\beta }$)	0.04413^{7}^	0.04556^{10}^	0.04143^{2}^	0.04168^{3}^	0.04449^{8}^	0.28434^{16}^	0.04375^{6}^	0.04102^{1}^	0.04314^{5}^	0.05112^{13}^	0.04951^{12}^	0.04536^{9}^	0.0423^{4}^	0.05638^{14}^	0.04566^{11}^	0.05913^{15}^
	BIAS($\overset{ˆ}{\gamma }$)	0.0068^{2}^	0.00699^{4}^	0.00794^{8}^	0.00664^{1}^	0.00808^{10}^	0.03572^{16}^	0.00715^{5}^	0.00698^{3}^	0.00799^{9}^	0.0082^{12}^	0.00751^{7}^	0.01316^{15}^	0.00811^{11}^	0.00901^{13}^	0.00716^{6}^	0.00957^{14}^
	MSE($\overset{ˆ}{\beta }$)	0.003^{6.5}^	0.00317^{9}^	0.0026^{1}^	0.00272^{3}^	0.00309^{8}^	0.12598^{16}^	0.003^{6.5}^	0.00261^{2}^	0.00287^{4}^	0.00436^{13}^	0.00398^{12}^	0.00326^{10}^	0.00299^{5}^	0.00513^{14}^	0.00339^{11}^	0.00544^{15}^
	MSE($\overset{ˆ}{\gamma }$)	7*e* − 05^{1.5}^	8*e* − 05^{4.5}^	1*e* − 04^{9.5}^	7*e* − 05^{1.5}^	1*e* − 04^{9.5}^	0.00192^{16}^	8*e* − 05^{4.5}^	8*e* − 05^{4.5}^	1*e* − 04^{9.5}^	0.00011^{12}^	9*e* − 05^{7}^	0.00029^{15}^	1*e* − 04^{9.5}^	0.00013^{13}^	8*e* − 05^{4.5}^	0.00014^{14}^
	MRE($\overset{ˆ}{\beta }$)	0.04904^{7}^	0.05062^{10}^	0.04603^{2}^	0.04631^{3}^	0.04944^{8}^	0.31593^{16}^	0.04861^{6}^	0.04558^{1}^	0.04793^{5}^	0.0568^{13}^	0.05501^{12}^	0.0504^{9}^	0.047^{4}^	0.06265^{14}^	0.05073^{11}^	0.0657^{15}^
	MRE($\overset{ˆ}{\gamma }$)	0.034^{2}^	0.03493^{4}^	0.0397^{8}^	0.03318^{1}^	0.04041^{10}^	0.17858^{16}^	0.03573^{5}^	0.03491^{3}^	0.03996^{9}^	0.04102^{12}^	0.03756^{7}^	0.0658^{15}^	0.04055^{11}^	0.04505^{13}^	0.0358^{6}^	0.04784^{14}^
	*D*_*abs*_	0.01306^{4.5}^	0.01353^{7}^	0.01303^{3}^	0.01252^{2}^	0.01365^{8}^	0.072^{16}^	0.01306^{4.5}^	0.01246^{1}^	0.0138^{9}^	0.01557^{12}^	0.01488^{11}^	0.02009^{15}^	0.01343^{6}^	0.017^{13}^	0.01388^{10}^	0.01799^{14}^
	*D*_*max*_	0.02106^{3}^	0.0218^{6}^	0.0216^{5}^	0.02017^{1}^	0.02245^{9}^	0.11181^{16}^	0.02114^{4}^	0.02029^{2}^	0.02285^{10}^	0.0252^{12}^	0.02387^{11}^	0.03594^{15}^	0.02228^{7}^	0.0274^{13}^	0.02239^{8}^	0.02904^{14}^
	ASAE	0.00369^{3}^	0.00439^{6}^	0.00481^{5}^	0.00361^{1}^	0.00506^{9}^	0.00313^{16}^	0.004^{4}^	0.00425^{2}^	0.00561^{10}^	0.00401^{12}^	0.00421^{11}^	0.02429^{15}^	0.00523^{7}^	0.00475^{13}^	0.00402^{8}^	0.00489^{14}^
	∑*Ranks*	45.5^{3}^	72.5^{7}^	47.5^{4}^	26.5^{1}^	81.5^{9}^	118^{16}^	54.5^{5}^	34.5^{2}^	73.5^{8}^	93^{11}^	95^{12}^	108^{14}^	69.5^{6}^	106^{13}^	82.5^{10}^	116^{15}^

**Table 5:** Numerical values of simulation measures for *β* = 4.0, *γ* = 2.5.

n	Est.	MLE	ADE	CVME	MPSE	OLSE	PCE	RTADE	WLSE	LTADE	MSADE	MSALDE	ADSOE	KE	MSSD	MSSLD	MSLND
20	BIAS($\overset{ˆ}{\beta }$)	0.81437^{11}^	0.75009^{6}^	0.8338^{12}^	0.69499^{2}^	0.79112^{9}^	0.7104^{4}^	0.73601^{5}^	0.79352^{10}^	0.90907^{15}^	0.70081^{3}^	0.7614^{8}^	1.11926^{16}^	0.53185^{1}^	0.87431^{13}^	0.7545^{7}^	0.87564^{14}^
	BIAS($\overset{ˆ}{\gamma }$)	0.37677^{2}^	0.38343^{3}^	0.45738^{13}^	0.39673^{5}^	0.42183^{9}^	0.37525^{1}^	0.41246^{8}^	0.40975^{7}^	0.42935^{10}^	0.43379^{11}^	0.44253^{12}^	0.47221^{14}^	0.38461^{4}^	0.51073^{15}^	0.40189^{6}^	0.52441^{16}^
	MSE($\overset{ˆ}{\beta }$)	1.21674^{14}^	1.00308^{8}^	1.21452^{13}^	0.74929^{2}^	1.00359^{9}^	0.84625^{3}^	0.98237^{6}^	1.04555^{10}^	1.43407^{15}^	0.8977^{4}^	0.91201^{5}^	1.99369^{16}^	0.62926^{1}^	1.11306^{12}^	1.00154^{7}^	1.10245^{11}^
	MSE($\overset{ˆ}{\gamma }$)	0.24239^{4}^	0.23945^{3}^	0.34412^{13}^	0.22693^{2}^	0.29017^{8}^	0.22321^{1}^	0.29443^{10}^	0.2724^{7}^	0.29801^{11}^	0.29945^{12}^	0.29222^{9}^	0.35834^{14}^	0.25279^{6}^	0.37622^{15}^	0.25209^{5}^	0.40689^{16}^
	MRE($\overset{ˆ}{\beta }$)	0.20359^{11}^	0.18752^{6}^	0.20845^{12}^	0.17375^{2}^	0.19778^{9}^	0.1776^{4}^	0.184^{5}^	0.19838^{10}^	0.22727^{15}^	0.1752^{3}^	0.19035^{8}^	0.27982^{16}^	0.13296^{1}^	0.21858^{13}^	0.18863^{7}^	0.21891^{14}^
	MRE($\overset{ˆ}{\gamma }$)	0.15071^{2}^	0.15337^{3}^	0.18295^{13}^	0.15869^{5}^	0.16873^{9}^	0.1501^{1}^	0.16498^{8}^	0.1639^{7}^	0.17174^{10}^	0.17351^{11}^	0.17701^{12}^	0.18888^{14}^	0.15385^{4}^	0.20429^{15}^	0.16075^{6}^	0.20976^{16}^
	*D*_*abs*_	0.05548^{1}^	0.05555^{3}^	0.0567^{5}^	0.05549^{2}^	0.06019^{10}^	0.05646^{4}^	0.0584^{8}^	0.05846^{9}^	0.05712^{6}^	0.06395^{13}^	0.06602^{14}^	0.06263^{12}^	0.05756^{7}^	0.07102^{15}^	0.0618^{11}^	0.07162^{16}^
	*D*_*max*_	0.09004^{5}^	0.08926^{4}^	0.09333^{7}^	0.08787^{1}^	0.09585^{10}^	0.08862^{2}^	0.09381^{9}^	0.09328^{6}^	0.09355^{8}^	0.10158^{12}^	0.10462^{14}^	0.10392^{13}^	0.08913^{3}^	0.11252^{15}^	0.09745^{11}^	0.11463^{16}^
	ASAE	0.04599^{5}^	0.04063^{4}^	0.04269^{7}^	0.04227^{1}^	0.04101^{10}^	0.04149^{2}^	0.0436^{9}^	0.04051^{6}^	0.04467^{8}^	0.05507^{12}^	0.05301^{14}^	0.05604^{13}^	0.04674^{3}^	0.05914^{15}^	0.0484^{11}^	0.06162^{16}^
	∑*Ranks*	59^{5}^	38^{4}^	94^{11.5}^	26^{2}^	76^{9}^	24^{1}^	66^{6}^	67^{7}^	98^{13}^	82^{10}^	94^{11.5}^	129^{15}^	37^{3}^	128^{14}^	71^{8}^	135^{16}^
80	BIAS($\overset{ˆ}{\beta }$)	0.37009^{4}^	0.3755^{6}^	0.44573^{12}^	0.35449^{3}^	0.42756^{11}^	0.35244^{2}^	0.37289^{5}^	0.38087^{7}^	0.52607^{15}^	0.40308^{9}^	0.41381^{10}^	0.78532^{16}^	0.28551^{1}^	0.502^{13}^	0.38923^{8}^	0.50214^{14}^
	BIAS($\overset{ˆ}{\gamma }$)	0.19491^{4}^	0.19397^{3}^	0.22106^{11}^	0.19321^{2}^	0.21884^{10}^	0.18106^{1}^	0.20052^{6}^	0.2065^{8}^	0.23125^{12}^	0.23284^{13}^	0.20882^{9}^	0.31546^{16}^	0.19525^{5}^	0.26894^{15}^	0.20147^{7}^	0.26589^{14}^
	MSE($\overset{ˆ}{\beta }$)	0.24228^{6}^	0.2306^{5}^	0.37241^{12}^	0.19398^{2}^	0.30147^{11}^	0.1981^{3}^	0.22533^{4}^	0.25407^{8}^	0.4829^{15}^	0.28098^{10}^	0.26814^{9}^	1.00618^{16}^	0.16993^{1}^	0.38942^{13}^	0.24697^{7}^	0.40567^{14}^
	MSE($\overset{ˆ}{\gamma }$)	0.06294^{5}^	0.06191^{3}^	0.07982^{11}^	0.05555^{2}^	0.07692^{10}^	0.05314^{1}^	0.06287^{4}^	0.0687^{9}^	0.08554^{12}^	0.09001^{13}^	0.06625^{8}^	0.15519^{16}^	0.06308^{6}^	0.11399^{15}^	0.06393^{7}^	0.10686^{14}^
	MRE($\overset{ˆ}{\beta }$)	0.09252^{4}^	0.09388^{6}^	0.11143^{12}^	0.08862^{3}^	0.10689^{11}^	0.08811^{2}^	0.09322^{5}^	0.09522^{7}^	0.13152^{15}^	0.10077^{9}^	0.10345^{10}^	0.19633^{16}^	0.07138^{1}^	0.1255^{13}^	0.09731^{8}^	0.12553^{14}^
	MRE($\overset{ˆ}{\gamma }$)	0.07796^{4}^	0.07759^{3}^	0.08843^{11}^	0.07728^{2}^	0.08754^{10}^	0.07242^{1}^	0.08021^{6}^	0.0826^{8}^	0.0925^{12}^	0.09314^{13}^	0.08353^{9}^	0.12618^{16}^	0.0781^{5}^	0.10758^{15}^	0.08059^{7}^	0.10636^{14}^
	*D*_*abs*_	0.02808^{2}^	0.02777^{1}^	0.02972^{7}^	0.02832^{3}^	0.03014^{9}^	0.02849^{4}^	0.03008^{8}^	0.02964^{6}^	0.03059^{10}^	0.03552^{13}^	0.03275^{12}^	0.03792^{15}^	0.02913^{5}^	0.03719^{14}^	0.03149^{11}^	0.03804^{16}^
	*D*_*max*_	0.04567^{4}^	0.04519^{1}^	0.04908^{8}^	0.0455^{3}^	0.04933^{9}^	0.04538^{2}^	0.04843^{7}^	0.04823^{6}^	0.05126^{11}^	0.0568^{13}^	0.05237^{12}^	0.06593^{16}^	0.04605^{5}^	0.06075^{14}^	0.05038^{10}^	0.06139^{15}^
	ASAE	0.01886^{4}^	0.01771^{1}^	0.01847^{8}^	0.01896^{3}^	0.01812^{9}^	0.01797^{2}^	0.01818^{7}^	0.01787^{6}^	0.01979^{11}^	0.02563^{13}^	0.0234^{12}^	0.02946^{16}^	0.01963^{5}^	0.02743^{14}^	0.02193^{10}^	0.02761^{15}^
	∑*Ranks*	40^{5}^	29^{3}^	90^{10}^	28^{2}^	85^{9}^	19^{1}^	50^{6}^	61^{7}^	112^{13}^	106^{12}^	91^{11}^	143^{16}^	38^{4}^	126^{14}^	76^{8}^	130^{15}^
150	BIAS($\overset{ˆ}{\beta }$)	0.26109^{4}^	0.28748^{7}^	0.32571^{12}^	0.2574^{3}^	0.30876^{11}^	0.25027^{2}^	0.292^{9}^	0.28475^{6}^	0.37346^{15}^	0.30836^{10}^	0.28751^{8}^	0.62293^{16}^	0.21653^{1}^	0.35375^{13}^	0.26985^{5}^	0.36184^{14}^
	BIAS($\overset{ˆ}{\gamma }$)	0.13759^{2}^	0.14902^{7}^	0.16163^{11}^	0.13782^{3}^	0.15832^{10}^	0.13045^{1}^	0.14988^{9}^	0.1458^{4}^	0.16695^{12}^	0.17121^{13}^	0.14972^{8}^	0.25124^{16}^	0.1481^{6}^	0.19242^{15}^	0.14711^{5}^	0.19092^{14}^
	MSE($\overset{ˆ}{\beta }$)	0.11471^{4}^	0.12937^{7}^	0.17893^{12}^	0.10302^{3}^	0.15241^{10}^	0.09366^{1}^	0.14493^{9}^	0.13188^{8}^	0.22765^{15}^	0.15957^{11}^	0.12847^{6}^	0.64216^{16}^	0.09825^{2}^	0.2078^{13}^	0.11503^{5}^	0.21002^{14}^
	MSE($\overset{ˆ}{\gamma }$)	0.0297^{3}^	0.03487^{7}^	0.04304^{11}^	0.02904^{2}^	0.04052^{10}^	0.02708^{1}^	0.03695^{9}^	0.03349^{5}^	0.04426^{12}^	0.04436^{13}^	0.035^{8}^	0.09824^{16}^	0.03377^{6}^	0.05859^{14}^	0.03347^{4}^	0.059^{15}^
	MRE($\overset{ˆ}{\beta }$)	0.06527^{4}^	0.07187^{7}^	0.08143^{12}^	0.06435^{3}^	0.07719^{11}^	0.06257^{2}^	0.073^{9}^	0.07119^{6}^	0.09336^{15}^	0.07709^{10}^	0.07188^{8}^	0.15573^{16}^	0.05413^{1}^	0.08844^{13}^	0.06746^{5}^	0.09046^{14}^
	MRE($\overset{ˆ}{\gamma }$)	0.05504^{2}^	0.05961^{7}^	0.06465^{11}^	0.05513^{3}^	0.06333^{10}^	0.05218^{1}^	0.05995^{9}^	0.05832^{4}^	0.06678^{12}^	0.06848^{13}^	0.05989^{8}^	0.1005^{16}^	0.05924^{6}^	0.07697^{15}^	0.05885^{5}^	0.07637^{14}^
	*D*_*abs*_	0.02^{1}^	0.02186^{7}^	0.02285^{10}^	0.02083^{3}^	0.02243^{8}^	0.02066^{2}^	0.02155^{5}^	0.02169^{6}^	0.0237^{12}^	0.02525^{13}^	0.02355^{11}^	0.02911^{16}^	0.02146^{4}^	0.028^{15}^	0.02246^{9}^	0.02727^{14}^
	*D*_*max*_	0.03244^{1}^	0.03529^{7}^	0.03746^{10}^	0.03341^{3}^	0.03651^{9}^	0.03294^{2}^	0.03507^{5}^	0.03512^{6}^	0.03923^{12}^	0.04088^{13}^	0.03768^{11}^	0.05157^{16}^	0.03425^{4}^	0.04548^{15}^	0.03607^{8}^	0.04455^{14}^
	ASAE	0.01306^{1}^	0.01236^{7}^	0.01282^{10}^	0.01287^{3}^	0.0127^{9}^	0.01269^{2}^	0.01269^{5}^	0.01236^{6}^	0.01346^{12}^	0.0177^{13}^	0.01624^{11}^	0.02235^{16}^	0.01374^{4}^	0.01925^{15}^	0.01532^{8}^	0.01918^{14}^
	∑*Ranks*	29^{2}^	58^{7}^	95^{11}^	30^{3}^	84^{10}^	15^{1}^	68^{8}^	46^{5}^	114^{13}^	109^{12}^	80^{9}^	144^{16}^	40^{4}^	128^{15}^	57^{6}^	127^{14}^
200	BIAS($\overset{ˆ}{\beta }$)	0.2253^{3}^	0.24435^{7}^	0.27019^{11}^	0.22024^{2}^	0.26851^{10}^	0.22579^{4}^	0.23244^{5}^	0.24084^{6}^	0.28956^{13}^	0.27472^{12}^	0.26326^{9}^	0.56749^{16}^	0.1844^{1}^	0.31369^{15}^	0.24784^{8}^	0.29706^{14}^
	BIAS($\overset{ˆ}{\gamma }$)	0.10868^{1}^	0.12828^{6}^	0.13933^{10}^	0.11286^{3}^	0.14077^{11}^	0.109^{2}^	0.12985^{8}^	0.12831^{7}^	0.14518^{12}^	0.15036^{13}^	0.13283^{9}^	0.22382^{16}^	0.11801^{4}^	0.16932^{15}^	0.12313^{5}^	0.16808^{14}^
	MSE($\overset{ˆ}{\beta }$)	0.08171^{4}^	0.09501^{7}^	0.12147^{12}^	0.07615^{2}^	0.1178^{10}^	0.07798^{3}^	0.08642^{5}^	0.09457^{6}^	0.13619^{13}^	0.12129^{11}^	0.10576^{9}^	0.51544^{16}^	0.06717^{1}^	0.14721^{15}^	0.09789^{8}^	0.14479^{14}^
	MSE($\overset{ˆ}{\gamma }$)	0.01936^{1}^	0.02571^{7}^	0.03091^{10}^	0.01943^{2}^	0.0319^{11}^	0.01957^{3}^	0.02659^{8}^	0.02553^{6}^	0.03364^{12}^	0.03447^{13}^	0.02728^{9}^	0.07897^{16}^	0.0225^{4}^	0.04616^{15}^	0.02467^{5}^	0.0443^{14}^
	MRE($\overset{ˆ}{\beta }$)	0.05633^{3}^	0.06109^{7}^	0.06755^{11}^	0.05506^{2}^	0.06713^{10}^	0.05645^{4}^	0.05811^{5}^	0.06021^{6}^	0.07239^{13}^	0.06868^{12}^	0.06582^{9}^	0.14187^{16}^	0.0461^{1}^	0.07842^{15}^	0.06196^{8}^	0.07427^{14}^
	MRE($\overset{ˆ}{\gamma }$)	0.04347^{1}^	0.05131^{6}^	0.05573^{10}^	0.04514^{3}^	0.05631^{11}^	0.0436^{2}^	0.05194^{8}^	0.05133^{7}^	0.05807^{12}^	0.06014^{13}^	0.05313^{9}^	0.08953^{16}^	0.0472^{4}^	0.06773^{15}^	0.04925^{5}^	0.06723^{14}^
	*D*_*abs*_	0.0174^{1}^	0.01802^{4}^	0.0191^{8}^	0.01805^{5}^	0.01944^{10}^	0.01777^{3}^	0.0183^{6}^	0.01841^{7}^	0.01915^{9}^	0.02208^{13}^	0.02183^{12}^	0.02629^{16}^	0.01745^{2}^	0.02373^{14}^	0.01977^{11}^	0.02459^{15}^
	*D*_*max*_	0.02803^{2}^	0.02943^{5}^	0.03138^{8}^	0.02887^{4}^	0.03182^{11}^	0.02842^{3}^	0.02984^{6}^	0.02992^{7}^	0.03168^{10}^	0.03574^{13}^	0.03479^{12}^	0.04691^{16}^	0.02772^{1}^	0.0387^{14}^	0.0316^{9}^	0.03967^{15}^
	ASAE	0.01089^{2}^	0.01036^{5}^	0.0107^{8}^	0.01103^{4}^	0.0106^{11}^	0.01081^{3}^	0.01068^{6}^	0.01046^{7}^	0.01129^{10}^	0.01473^{13}^	0.01382^{12}^	0.01939^{16}^	0.01143^{1}^	0.01648^{14}^	0.01306^{9}^	0.01652^{15}^
	∑*Ranks*	23^{1}^	50^{5}^	85^{9}^	31^{4}^	87^{10}^	30^{3}^	55^{7}^	54^{6}^	103^{12}^	113^{13}^	90^{11}^	144^{16}^	28^{2}^	132^{15}^	70^{8}^	129^{14}^
300	BIAS($\overset{ˆ}{\beta }$)	0.17196^{2}^	0.19649^{6}^	0.22709^{12}^	0.18099^{4}^	0.2208^{11}^	0.1809^{3}^	0.19103^{5}^	0.19821^{8}^	0.24883^{14}^	0.21731^{10}^	0.20958^{9}^	0.46025^{16}^	0.16171^{1}^	0.26108^{15}^	0.19773^{7}^	0.24596^{13}^
	BIAS($\overset{ˆ}{\gamma }$)	0.09578^{2}^	0.09778^{4}^	0.11582^{10}^	0.09848^{5}^	0.11585^{11}^	0.09458^{1}^	0.10674^{8}^	0.09684^{3}^	0.11733^{12}^	0.12032^{13}^	0.10926^{9}^	0.18495^{16}^	0.10456^{7}^	0.13362^{14}^	0.10368^{6}^	0.14004^{15}^
	MSE($\overset{ˆ}{\beta }$)	0.04842^{1}^	0.06176^{7}^	0.08723^{12}^	0.05081^{3}^	0.07601^{10}^	0.05078^{2}^	0.05807^{5}^	0.06232^{8}^	0.10083^{14}^	0.07754^{11}^	0.06926^{9}^	0.34296^{16}^	0.05203^{4}^	0.10735^{15}^	0.06068^{6}^	0.09816^{13}^
	MSE($\overset{ˆ}{\gamma }$)	0.01445^{2}^	0.01525^{4}^	0.02112^{10}^	0.01507^{3}^	0.02116^{11}^	0.01399^{1}^	0.01789^{8}^	0.01539^{5}^	0.02203^{12}^	0.02253^{13}^	0.01854^{9}^	0.05378^{16}^	0.01773^{7}^	0.02806^{14}^	0.01713^{6}^	0.03021^{15}^
	MRE($\overset{ˆ}{\beta }$)	0.04299^{2}^	0.04912^{6}^	0.05677^{12}^	0.04525^{4}^	0.0552^{11}^	0.04522^{3}^	0.04776^{5}^	0.04955^{8}^	0.06221^{14}^	0.05433^{10}^	0.0524^{9}^	0.11506^{16}^	0.04043^{1}^	0.06527^{15}^	0.04943^{7}^	0.06149^{13}^
	MRE($\overset{ˆ}{\gamma }$)	0.03831^{2}^	0.03911^{4}^	0.04633^{10}^	0.03939^{5}^	0.04634^{11}^	0.03783^{1}^	0.0427^{8}^	0.03874^{3}^	0.04693^{12}^	0.04813^{13}^	0.04371^{9}^	0.07398^{16}^	0.04183^{7}^	0.05345^{14}^	0.04147^{6}^	0.05602^{15}^
	*D*_*abs*_	0.01418^{1}^	0.01476^{4}^	0.01536^{9}^	0.01472^{3}^	0.01527^{8}^	0.01468^{2}^	0.01508^{6}^	0.01497^{5}^	0.0157^{10}^	0.0178^{13}^	0.01681^{12}^	0.02135^{16}^	0.01517^{7}^	0.01983^{14}^	0.01622^{11}^	0.02036^{15}^
	*D*_*max*_	0.02294^{1}^	0.02392^{4}^	0.0254^{9}^	0.02379^{3}^	0.02515^{8}^	0.02361^{2}^	0.02448^{7}^	0.02414^{5}^	0.0262^{11}^	0.02882^{13}^	0.02706^{12}^	0.03835^{16}^	0.02437^{6}^	0.03218^{14}^	0.02597^{10}^	0.03293^{15}^
	ASAE	0.0086^{1}^	0.00823^{4}^	0.00854^{9}^	0.00862^{3}^	0.00851^{8}^	0.00838^{2}^	0.00852^{7}^	0.00827^{5}^	0.00894^{11}^	0.01212^{13}^	0.01102^{12}^	0.01579^{16}^	0.00916^{6}^	0.0132^{14}^	0.01014^{10}^	0.0133^{15}^
	∑*Ranks*	20^{2}^	40^{4}^	90^{10.5}^	38^{3}^	85^{9}^	18^{1}^	57^{7}^	47^{5}^	108^{12}^	109^{13}^	90^{10.5}^	144^{16}^	50^{6}^	129^{14.5}^	70^{8}^	129^{14.5}^
400	BIAS($\overset{ˆ}{\beta }$)	0.15478^{3}^	0.16535^{6}^	0.1952^{12}^	0.16108^{5}^	0.18232^{9}^	0.15133^{2}^	0.16017^{4}^	0.1741^{8}^	0.21142^{13}^	0.19332^{11}^	0.18449^{10}^	0.41139^{16}^	0.13668^{1}^	0.22395^{15}^	0.16859^{7}^	0.21813^{14}^
	BIAS($\overset{ˆ}{\gamma }$)	0.08195^{1}^	0.08724^{5}^	0.09865^{11}^	0.08408^{2}^	0.09632^{10}^	0.08482^{3}^	0.09549^{9}^	0.09065^{7}^	0.09986^{12}^	0.10577^{13}^	0.08997^{6}^	0.15809^{16}^	0.09123^{8}^	0.12103^{15}^	0.08711^{4}^	0.11532^{14}^
	MSE($\overset{ˆ}{\beta }$)	0.03905^{3}^	0.04398^{7}^	0.05933^{11}^	0.03946^{4}^	0.05366^{10}^	0.03688^{1}^	0.0421^{5}^	0.04717^{8}^	0.07035^{13}^	0.0607^{12}^	0.05355^{9}^	0.26847^{16}^	0.0378^{2}^	0.07866^{15}^	0.04387^{6}^	0.07514^{14}^
	MSE($\overset{ˆ}{\gamma }$)	0.01094^{2}^	0.01206^{4}^	0.01569^{11}^	0.01066^{1}^	0.01486^{10}^	0.01095^{3}^	0.01423^{9}^	0.01269^{6}^	0.01596^{12}^	0.01752^{13}^	0.01285^{7}^	0.04088^{16}^	0.01395^{8}^	0.02268^{15}^	0.01231^{5}^	0.02096^{14}^
	MRE($\overset{ˆ}{\beta }$)	0.0387^{3}^	0.04134^{6}^	0.0488^{12}^	0.04027^{5}^	0.04558^{9}^	0.03783^{2}^	0.04004^{4}^	0.04352^{8}^	0.05286^{13}^	0.04833^{11}^	0.04612^{10}^	0.10285^{16}^	0.03417^{1}^	0.05599^{15}^	0.04215^{7}^	0.05453^{14}^
	MRE($\overset{ˆ}{\gamma }$)	0.03278^{1}^	0.0349^{5}^	0.03946^{11}^	0.03363^{2}^	0.03853^{10}^	0.03393^{3}^	0.0382^{9}^	0.03626^{7}^	0.03994^{12}^	0.04231^{13}^	0.03599^{6}^	0.06324^{16}^	0.03649^{8}^	0.04841^{15}^	0.03484^{4}^	0.04613^{14}^
	*D*_*abs*_	0.01258^{1}^	0.01273^{2}^	0.01347^{7}^	0.01286^{3}^	0.01385^{9}^	0.01298^{4}^	0.01373^{8}^	0.01312^{6}^	0.01386^{10}^	0.01601^{13}^	0.01459^{12}^	0.01945^{16}^	0.01299^{5}^	0.01779^{15}^	0.01401^{11}^	0.01725^{14}^
	*D*_*max*_	0.0202^{1}^	0.02062^{2}^	0.02217^{7}^	0.02069^{3}^	0.02253^{10}^	0.02076^{4}^	0.02218^{8}^	0.02132^{6}^	0.02297^{11}^	0.02579^{13}^	0.02347^{12}^	0.03449^{16}^	0.02085^{5}^	0.02885^{15}^	0.02246^{9}^	0.02789^{14}^
	ASAE	0.00724^{1}^	0.00702^{2}^	0.00722^{7}^	0.00741^{3}^	0.00712^{10}^	0.00715^{4}^	0.00715^{8}^	0.00692^{6}^	0.00757^{11}^	0.01001^{13}^	0.00939^{12}^	0.01357^{16}^	0.00789^{5}^	0.01123^{15}^	0.00877^{9}^	0.01102^{14}^
	∑*Ranks*	22^{1}^	39^{4}^	88^{11}^	33^{3}^	80^{9}^	26^{2}^	61^{7}^	57^{6}^	105^{12}^	112^{13}^	84^{10}^	144^{16}^	48^{5}^	135^{15}^	64^{8}^	126^{14}^

**Table 6:** Partial and overall ranks of all the methods of estimating proposed distribution by various values of model parameters.

Parameter	*n*	MLE	ADE	CVME	MPSE	OLSE	PCE	RTADE	WLSE	LTADE	MSADE	MSALDE	ADSOE	KE	MSSD	MSSLD	MSLND
*β* = 2.5, *γ* = 1.5	20	5.0	2.0	13.0	3.0	7.0	1.0	8.0	6.0	12.0	10.0	9.0	16.0	11.0	14.0	4.0	15.0
	80	5.0	1.0	8.0	2.0	9.0	4.0	3.0	6.0	13.0	12.0	11.0	16.0	10.0	15.0	7.0	14.0
	150	1.0	4.0	10.0	2.0	9.0	6.0	3.0	5.0	12.0	13.0	11.0	16.0	7.0	15.0	8.0	14.0
	200	1.0	3.5	10.0	2.0	7.0	3.5	5.0	6.0	12.0	13.0	11.0	16.0	8.0	15.0	9.0	14.0
	300	1.0	3.0	11.0	2.0	10.0	5.0	6.0	4.0	12.0	13.0	7.0	16.0	9.0	14.0	8.0	15.0
	400	1.0	3.0	12.0	2.0	7.0	6.0	5.0	4.0	11.0	13.0	10.0	16.0	8.0	15.0	9.0	14.0

*β* = 0.5, *γ* = 0.3	20	4.0	7.5	10.0	3.0	7.5	2.0	11.0	6.0	12.5	5.0	12.5	14.0	1.0	16.0	9.0	15.0
	80	2.0	4.0	11.0	3.0	8.0	5.0	7.0	6.0	12.0	9.0	13.0	14.0	1.0	15.0	10.0	16.0
	150	3.0	6.0	8.0	2.0	9.0	4.0	7.0	5.0	11.0	13.0	12.0	14.5	1.0	16.0	10.0	14.5
	200	3.0	4.0	11.0	5.0	8.0	1.0	7.0	6.0	9.0	13.0	12.0	14.0	2.0	15.0	10.0	16.0
	300	3.0	4.0	7.0	2.0	8.0	5.0	9.0	6.0	11.0	13.0	12.0	14.0	1.0	15.5	10.0	15.5
	400	5.0	4.0	10.0	3.0	8.0	1.0	7.0	6.0	9.0	13.0	12.0	14.0	2.0	16.0	11.0	15.0

*β* = 2.0, *γ* = 0.7	20	4.0	3.0	9.0	1.0	11.0	13.0	5.0	2.0	14.0	7.0	10.0	16.0	8.0	15.0	6.0	12.0
	80	2.0	3.0	9.0	1.0	7.0	13.0	5.0	6.0	11.0	12.0	8.0	16.0	10.0	15.0	4.0	14.0
	150	1.0	3.0	8.0	2.0	7.0	13.0	5.0	4.0	9.5	12.0	11.0	16.0	9.5	15.0	6.0	14.0
	200	2.0	3.0	9.0	1.0	6.5	13.0	4.0	5.0	11.0	12.0	10.0	16.0	6.5	14.0	8.0	15.0
	300	1.0	3.0	7.0	2.0	6.0	13.0	5.0	4.0	11.0	12.0	10.0	16.0	9.0	15.0	8.0	14.0
	400	1.0	4.0	7.0	2.0	6.0	14.0	3.0	5.0	10.0	12.0	11.0	16.0	9.0	13.0	8.0	15.0

*β* = 0.9, *γ* = 0.2	20	1.0	3.0	10.0	2.0	11.0	15.0	7.0	4.0	13.0	6.0	9.0	12.0	5.0	16.0	8.0	14.0
	80	1.0	2.0	8.0	4.0	10.0	16.0	6.0	3.0	7.0	12.0	11.0	13.0	5.0	14.0	9.0	15.0
	150	2.0	3.0	9.0	1.0	5.0	16.0	4.0	7.0	11.0	12.0	10.0	13.0	6.0	15.0	8.0	14.0
	200	1.0	2.0	11.0	3.0	10.0	16.0	6.0	4.0	7.0	12.0	9.0	13.0	5.0	15.0	8.0	14.0
	300	1.0	8.0	3.0	4.0	5.0	16.0	7.0	2.0	6.0	12.0	9.0	13.0	10.0	14.0	11.0	15.0
	400	3.0	7.0	4.0	1.0	9.0	16.0	5.0	2.0	8.0	11.0	12.0	14.0	6.0	13.0	10.0	15.0

*β* = 4.0, *γ* = 2.5	20	5.0	4.0	11.5	2.0	9.0	1.0	6.0	7.0	13.0	10.0	11.5	15.0	3.0	14.0	8.0	16.0
	80	5.0	3.0	10.0	2.0	9.0	1.0	6.0	7.0	13.0	12.0	11.0	16.0	4.0	14.0	8.0	15.0
	150	2.0	7.0	11.0	3.0	10.0	1.0	8.0	5.0	13.0	12.0	9.0	16.0	4.0	15.0	6.0	14.0
	200	1.0	5.0	9.0	4.0	10.0	3.0	7.0	6.0	12.0	13.0	11.0	16.0	2.0	15.0	8.0	14.0
	300	2.0	4.0	10.5	3.0	9.0	1.0	7.0	5.0	12.0	13.0	10.5	16.0	6.0	14.5	8.0	14.5
	400	1.0	4.0	11.0	3.0	9.0	2.0	7.0	6.0	12.0	13.0	10.0	16.0	5.0	15.0	8.0	14.0

∑ Ranks		70.0	117.0	278.0	72.0	247.0	226.5	181.0	150.0	330.0	345.0	315.5	449.5	174.0	443.0	245.0	436.5
Overall Rank		1	3	10	2	9	7	6	4	12	13	11	16	5	15	8	14

## 8 Real data analysis

In this section, we provide two practical data examples to prove the usefulness of the PNXL distribution in modeling real-world data. We consider selection criteria, namely, −2×log⁡(ℓ), Akaike's information criterion (AIC), Kolmogorov-Smirnov (KS), Anderson-Darling (AD), Cramer-von Mises (CvM) test statistics and their p-values to compare the fitted distributions. We choose the following competitors to PNXL distribution, NXL [12], XLindley (XL) [7], Lindley (L) [8], Akash (A) [14], Shanker (S) [15], Exponential Power (EP) [22], exponential (E) distributions. The PDFs of the fitted distributions are given in Table 7.

**Table 7:** List of the lifetime distribution to modeling datasets.

$f_{NXL}\left(x;\gamma \right)=\frac{\gamma }{2}\left(1+\gamma x\right)\exp\left(-\gamma x\right)$	,	*γ* > 0
$f_{XL}\left(x;\gamma \right)=\frac{\gamma^{2}\left(\gamma +x+2\right)\exp\left(-\gamma x\right)}{{\left(1+\gamma \right)}^{2}}$	,	*γ* > 0
$f_{L}\left(x;\gamma \right)=\frac{\gamma^{2}\left(x+1\right)\exp\left(-\gamma x\right)}{1+\gamma }$	,	*γ* > 0
$f_{EP}\left(x\right)=\frac{\gamma }{\beta }{\left(\frac{x}{\beta }\right)}^{\gamma -1}e^{{\left(\frac{x}{\beta }\right)}^{\gamma }}e^{\left[1-e^{{\left(\frac{x}{\beta }\right)}^{\gamma }}\right]}$	,	*γ*,*β* > 0
$f_{A}\left(x;\gamma \right)=\frac{\gamma^{3}\left(1+x^{2}\right)\exp\left(-\gamma x\right)}{\gamma^{2}+2}$	,	*γ* > 0
$f_{S}\left(x;\gamma \right)=\frac{\gamma^{2}\left(\gamma +x\right)\exp\left(-\gamma x\right)}{\gamma^{2}+1}$	,	*γ* > 0
$f_{E}\left(x;\gamma \right)=\gamma \exp\left(-\gamma x\right)$	,	*γ* > 0

### 8.1 Data set 1

**Figure 7:**
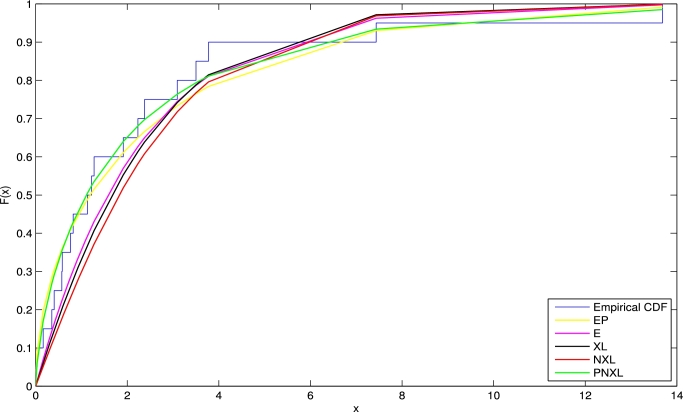
The fitted CDFs for the first data set.

**Figure 8:**
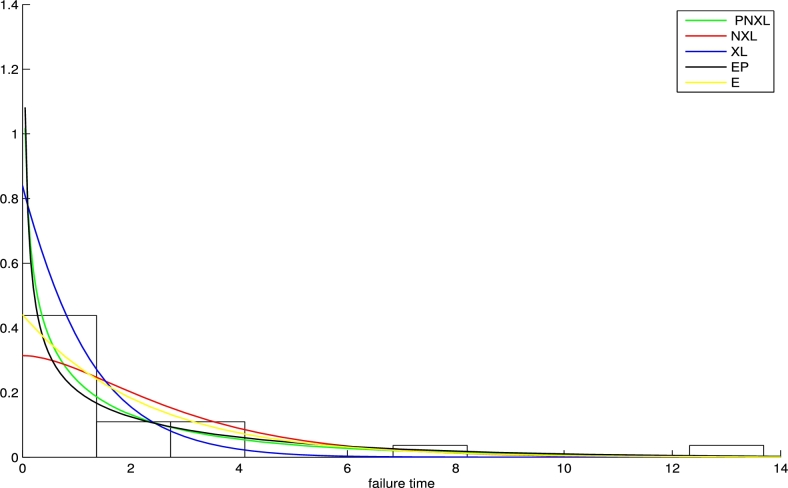
The fitted PDFs for the first data set.

The first dataset includes 20 observations developed by [11]. The right-skewed data represents the failure times of 20 components. The data are: 0.0003, 0.0298, 0.1648, 0.3529, 0.4044, 0.5712, 0.5808, 0.7607, 0.8188, 1.1296, 1.2228, 1.2773, 1.9115, 2.2333, 2.3791, 3.0916, 3.4999, 3.7744, 7.4339, 13.6866. Table 8 provides the MLEs and corresponding standard errors (SEs) of the parameters of the fitted distributions, while Table 9 shows the selection criteria for flood peaks data. Also, Figure 7, Figure 8 illustrate that the fitted CDFs and PDFs for the first data set, respectively.

**Table 8:** The MLEs and SEs of the parameters of fitted distributions for the first data set.

Distribution	$\overset{ˆ}{\gamma }$	$\overset{ˆ}{\beta }$	SE$\left(\overset{ˆ}{\gamma }\right)$	SE$\left(\overset{ˆ}{\beta }\right)$
PNXL	0.6270	1.0746	0.1124	0.2558
NXL	0.6285	-	0.1260	-
XL	0.6152	-	0.1044	-
L	0.5818	-	0.1148	-
EP	0.4924	4.3805	0.0882	1.2311
A	1.0213	-	0.1274	-
S	0.7565	-	0.1077	-
E	0.4413	-	0.0987	-

**Table 9:** The selection criteria for the first data set.

	−2log⁡ℓ	AIC	KS	AD	CvM	p-value (KS)	p-value (AD)	p-value (CvM)
PNXL	67.1956	71.1956	0.1185	0.2903	0.0374	0.9105	0.9447	0.9502
NXL	75.8746	77.8746	0.2279	1.8380	0.2492	0.2143	0.1135	0.1897
XL	74.6916	76.6916	0.1930	1.3300	0.1579	0.3949	0.2226	0.3686
L	78.3507	80.3507	0.2991	3.3534	0.5233	0.0441	0.0185	0.0337
EP	67.5783	71.5783	0.1345	0.3848	0.0595	0.8160	0.8622	0.8229
A	81.1626	83.1626	0.2556	2.3025	0.3026	0.1221	0.0636	0.1325
S	78.1524	80.1524	0.2143	1.8479	0.2140	0.2757	0.1121	0.2428
E	72.7239	74.7239	0.1691	0.9941	0.1104	0.5597	0.3593	0.5401

Figure 7, Figure 8 illustrate the superiority of the PNXL distribution over its competitors in modeling the first data set. From Table 9, we concluded that the PNXL distribution is the best-fitted model for the first data set according to all comparison criteria. As a result of the first data analysis, we concluded that the PNXL distribution is a good rival to the NXL distribution in real-world data modeling.

### 8.2 Data set 2

**Figure 9:**
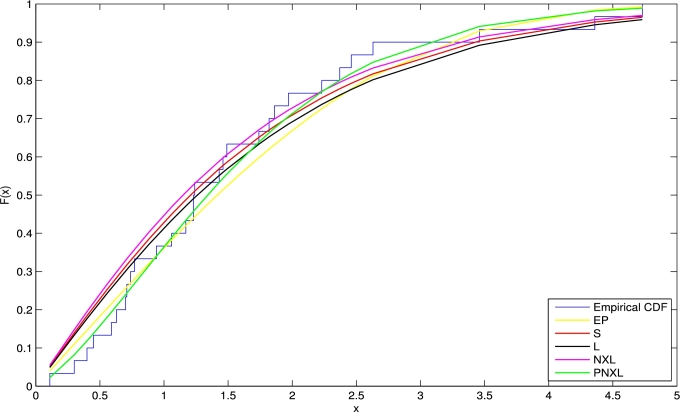
The fitted CDFs for the second data set.

**Figure 10:**
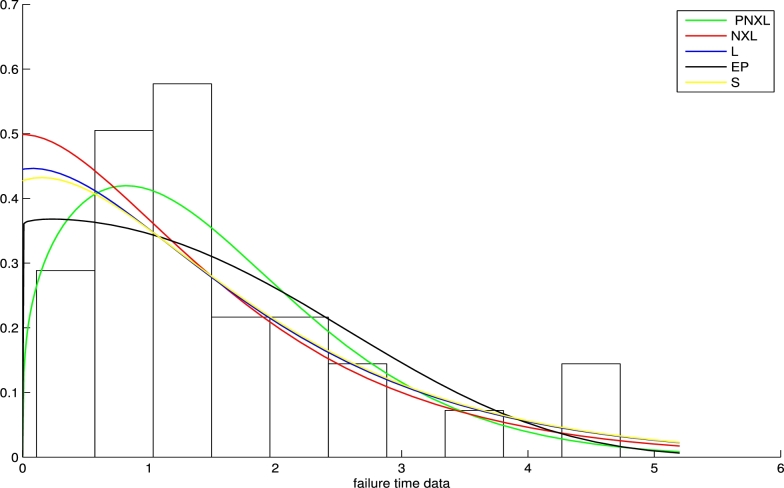
The fitted PDFs for the second data set.

The second dataset consists of 20 observations on the time between failures for repairable items, discussed by [11]. The second data set is 1.43, 0.11, 0.71, 0.77, 2.63, 1.49, 3.46, 2.46, 0.59, 0.74, 1.23, 0.94, 4.36, 0.40, 1.74, 4.73, 2.23, 0.45, 0.70, 1.06, 1.46, 0.30, 1.82, 2.37, 0.63, 1.23, 1.24, 1.97, 1.86, 1.17. Table 10 presents the MLEs and corresponding standard errors of the parameters of the fitted distributions. On the other hand, Table 11 provides the selection criteria for the second data set. Also, Figure 9, Figure 10 illustrate the fitted CDFs and PDFs for the second data set.

**Table 10:** The MLEs and SEs of the parameters of fitted distributions for the second data set.

Distribution	$\overset{ˆ}{\gamma }$	$\overset{ˆ}{\beta }$	SE$\left(\overset{ˆ}{\gamma }\right)$	SE$\left(\overset{ˆ}{\beta }\right)$
PNXL	1.2959	0.7840	0.1840	0.1782
NXL	0.9976	-	0.1585	-
XL	0.8365	-	0.1197	-
L	0.9258	-	0.1396	-
EP	1.0076	2.6779	0.1471	0.3023
A	1.3342	-	0.1394	-
S	0.9649	-	0.1169	-
E	0.6482	-	0.1183	-

**Table 11:** The selection criteria for the second data set.

	−2log⁡ℓ	AIC	KS	AD	CvM	p-value (KS)	p-value (AD)	p-value (CvM)
PNXL	80.1895	84.1895	0.0785	0.2327	0.0303	0.9926	0.9787	0.9769
NXL	83.1250	85.1250	0.1482	0.7990	0.1301	0.5253	0.4806	0.4585
XL	84.8704	86.8704	0.1669	1.0646	0.1777	0.3736	0.3245	0.3162
L	83.2408	85.2408	0.1233	0.6524	0.0963	0.7522	0.5985	0.6067
EP	82.7638	86.7638	0.1104	0.5640	0.0863	0.8580	0.6813	0.6596
A	84.2483	86.2483	0.1474	0.8257	0.1269	0.5325	0.4618	0.4706
S	83.0375	85.0375	0.1339	0.6755	0.1028	0.6553	0.5783	0.5743
E	86.0108	88.0108	0.1845	1.3298	0.2324	0.2590	0.2228	0.2129

Figure 9, Figure 10 show the superiority of the PNXL distribution over its competitors in modeling the second data set. Table 11 shows that the PNXL distribution is the best-fitted model for the second data set according to all comparison criteria. As a result of the goodness-of-fit test for the first and second data, the PNXL distribution was found to be a worthy alternative to the NXL distributions and other competitors.

## 9 Conclusion

In this section, we discuss the conclusions of this study. We propose a new statistical distribution called the PNXL distribution as an alternative to NXL and other competitors. The hazard function of PNXL distribution can decrease or increase, and the bathtub is curve-shaped. For this reason, we recommend using the PNXL distribution in modeling data. We suggest sixteen estimators of the parameters of the PNXL distribution. We design a comprehensive Monte Carlo simulation study to assess the performances of the suggested estimators. As a result of the simulation study, we have observed the following results. When β=2.5,γ=1.5, ADE is the best estimator for small sample sizes, and MLE is the best for large sample sizes. KE is the best estimator when β=0.5,γ=0.3. When β=2,γ=0.7, MPSE is the best predictor in small sample sizes, and MLE is the best predictor in large sample sizes. When β=0.9,γ=0.2, MLE and MPSE are the two best estimators. Also, we provide two real-world data examples to compare the fits of the well-known lifetime distributions. According to practical data results, the PNXL distribution is the best-fitted distribution for the first data set. Similarly, in the second data analysis, the PNXL distribution is the best-fitted model according to all selection criteria. In conclusion, we suggest a new distribution of the literature as an alternative to the modified ones and the NXL.

In future studies, we believe this study will inspire the proposal of new distributions that can be alternatives to this distribution. Future research could delve deeper into the theoretical properties of the PNXL distribution, examining its behavior under various transformations and its potential for generating new distribution families. Expanding comparative studies with other distributions across broader applications would offer additional insights into its practical utility. Developing computational tools and software packages for the PNXL distribution would also enhance its accessibility and application in various fields.

## CRediT authorship contribution statement

**Ahmed M. Gemeay:** Writing – review & editing, Validation, Software, Project administration, Investigation, Data curation, Conceptualization. **Abdelali Ezzebsa:** Writing – review & editing, Validation, Supervision, Formal analysis, Conceptualization. **Halim Zeghdoudi:** Writing – original draft, Visualization, Validation, Methodology, Data curation. **Caner Tanış:** Writing – original draft, Visualization, Resources, Formal analysis. **Yusra A. Tashkandy:** Writing – review & editing, Writing – original draft, Methodology, Formal analysis. **M.E. Bakr:** Writing – review & editing, Writing – original draft, Supervision, Resources, Data curation, Conceptualization. **Anoop Kumar:** Writing – original draft, Validation, Resources, Methodology, Data curation, Conceptualization.

## Declaration of Competing Interest

The authors declare that they have no known competing financial interests or personal relationships that could have appeared to influence the work reported in this paper.

## Data Availability

The data that supports the findings of this study are available within the article.

## References

[1] Aguilar G.A., Moala F.A., Cordeiro G.M. (2019). Zero-truncated Poisson exponentiated gamma distribution: application and estimation methods. J. Stat. Theory Pract..

[2] Aidi K., Al-Omari A.I., Alsultan R. (2022). The power zeghdoudi distribution: properties, estimation, and applications to real right-censored data. Appl. Sci..

[3] Anderson T.W., Darling D.A. (1952). Asymptotic theory of certain “goodness of fit” criteria based on stochastic processes. Ann. Math. Stat..

[4] Benatmane C., Zeghdoudi H., Ezzebsa A., Bouchahed L. (2021). Note on quasi Lindley distribution: some remarks and corrections. Asian J. Probab. Stat..

[5] Cheng R., Amin N. (1983). Estimating parameters in continuous univariate distributions with a shifted origin. J. R. Stat. Soc., Ser. B, Methodol..

[6] Choi K., Bulgren W.G. (1968). An estimation procedure for mixtures of distributions. J. R. Stat. Soc., Ser. B, Methodol..

[7] Chouia S., Zeghdoudi H. (2021). The xlindley distribution: properties and application. J. Stat. Theory Appl..

[8] Ghitany M.E., Atieh B., Nadarajah S. (2008). Lindley distribution and its application. Math. Comput. Simul..

[9] Kao J.H. (1958). Computer methods for estimating Weibull parameters in reliability studies. IRE Trans. Reliab. Qual. Control.

[10] Kao J.H. (1959). A graphical estimation of mixed Weibull parameters in life-testing of electron tubes. Technometrics.

[11] Murthy D.P., Xie M., Jiang R. (2004).

[12] Nawel K., Gemeay A.M., Zeghdoudi H., Karakaya K., Alshangiti A.M., Bakr M.E., Balogun O.S., Muse A.H., Hussam E. (2023). Modeling voltage real data set by a new version of Lindley distribution. IEEE Access.

[13] Nedjar S., Zeghdoudi H. (2016). On gamma Lindley distribution: properties and simulations. J. Comput. Appl. Math..

[14] (2015). Int. J. Probab. Stat..

[15] Shanker R. (2015). Shanker distribution and its applications. Int. J. Stat. Appl..

[16] Shanker R., Amanuel A.G. (2013). A new quasi Lindley distribution. Int. J. Stat. Syst..

[17] Shanker R., Sharma S., Shanker R. (2013). A two-parameter Lindley distribution for modeling waiting and survival times data. Appl. Math..

[18] Shanker R., Shukla K.K. (2017). Power shanker distribution and its application. Türkiye Klinikleri Biyoistatistik.

[19] Shanker R., Shukla K.K. (2018). A two-parameter power aradhana distribution with properties and application. Indian Soc. Indust. Appl. Math..

[20] Shanker R., Shukla K.K. (2019). A two-parameter power sujatha distribution with properties and application. Int. J. Math. Stat..

[21] Shukla K.K., Shanker R. (2020). Power prakaamy distribution and its applications. Int. J. Comput. Theor. Stat..

[22] Smith R.M., Bain L.J. (1975). An exponential power life-testing distribution. Commun. Stat., Theory Methods.

[23] Swain J.J., Venkatraman S., Wilson J.R. (1988). Least-squares estimation of distribution functions in Johnson's translation system. J. Stat. Comput. Simul..

[24] Torabi H. (2008). A general method for estimating and hypotheses testing using spacings. J. Stat. Theory Appl..

[25] Zeghdoudi H., Nedjar S. (2016). Gamma Lindley distribution and its application. J. Appl. Probab. Stat..

[26] Zeghdoudi H., Nedjar S. (2016). A pseudo Lindley distribution and its application. Afr. Stat..

